# Regulation of Sulphur Assimilation Is Essential for Virulence and Affects Iron Homeostasis of the Human-Pathogenic Mould *Aspergillus fumigatus*

**DOI:** 10.1371/journal.ppat.1003573

**Published:** 2013-08-29

**Authors:** Jorge Amich, Lukas Schafferer, Hubertus Haas, Sven Krappmann

**Affiliations:** 1 Research Center for Infectious Diseases, Julius-Maximilians-University Würzburg, Würzburg, Germany; 2 Division of Molecular Biology/Biocenter, Innsbruck Medical University, Innsbruck, Austria; 3 Mikrobiologisches Institut - Klinische Mikrobiologie, Immunologie und Hygiene, Universitätsklinikum Erlangen, Friedrich-Alexander-Universität Erlangen-Nürnberg, Erlangen, Germany; Albert Einstein College of Medicine, United States of America

## Abstract

Sulphur is an essential element that all pathogens have to absorb from their surroundings in order to grow inside their infected host. Despite its importance, the relevance of sulphur assimilation in fungal virulence is largely unexplored. Here we report a role of the bZIP transcription factor MetR in sulphur assimilation and virulence of the human pathogen *Aspergillus fumigatus*. The MetR regulator is essential for growth on a variety of sulphur sources; remarkably, it is fundamental for assimilation of inorganic S-sources but dispensable for utilization of methionine. Accordingly, it strongly supports expression of genes directly related to inorganic sulphur assimilation but not of genes connected to methionine metabolism. On a broader scale, MetR orchestrates the comprehensive transcriptional adaptation to sulphur-starving conditions as demonstrated by digital gene expression analysis. Surprisingly, *A. fumigatus* is able to utilize volatile sulphur compounds produced by its methionine catabolism, a process that has not been described before and that is MetR-dependent. The *A. fumigatus* MetR transcriptional activator is important for virulence in both leukopenic mice and an alternative mini-host model of aspergillosis, as it was essential for the development of pulmonary aspergillosis and supported the systemic dissemination of the fungus. MetR action under sulphur-starving conditions is further required for proper iron regulation, which links regulation of sulphur metabolism to iron homeostasis and demonstrates an unprecedented regulatory crosstalk. Taken together, this study provides evidence that regulation of sulphur assimilation is not only crucial for *A. fumigatus* virulence but also affects the balance of iron in this prime opportunistic pathogen.

## Author Summary

Invasive pulmonary aspergillosis (IPA) is a life-threatening disease that affects primarily immunosuppressed patients. During the last decades the incidence of this disease that is accompanied by high mortality rates has increased. Since opportunistic pathogenic fungi, unlike other pathogens, do not express specific virulence factors, it is becoming more and more clear that the elucidation of fungal metabolism is an essential task to understand fungal pathogenicity and to identify novel antifungal targets. In this work we report genetic inactivation of the sulphur transcription regulator MetR in *Aspergillus fumigatus* and subsequent study of the resulting phenotypes and transcriptional deregulation of the mutant. Here we show that regulation of sulphur assimilation is an essential process for the manifestation of IPA. Moreover, a regulatory connection between sulphur metabolism and iron homeostasis, a further essential virulence determinant of *A. fumigatus*, is demonstrated in this study for the first time. A deeper knowledge of sulphur metabolism holds the promise of increasing our understanding of fungal virulence and might lead to improved antifungal therapy.

## Introduction

*Aspergillus fumigatus* is an opportunistic fungal pathogen that may cause invasive infections in immunocompromised patients. During the last decades the incidence rate of invasive pulmonary aspergillosis (IPA), the most severe infection caused by this mould [1], has dramatically increased which mainly results from the rise in immune-suppressive medical therapies. Furthermore, IPA is accompanied by a high mortality rate which may reach up to 90% depending on the immune status of the host [2], [3], [4]. This pronounced lethality is primarily attributed to the relative inefficiency of current chemotherapies [5], which are based on disrupting the integrity of the fungal cell membrane or cell wall [6]. Therefore, an urgent need to identify targets for the development of novel antifungal substances is evident.

Nutritional supply is an essential prerequisite for the onset and manifestation of infection by any pathogen [7]. In opportunistic fungi like *A. fumigatus*, which does not seem to express host-specific virulence factors [8], [9], nutrient uptake and metabolic versatility have to be considered as non-specific virulence determinants (for a recent review see [10]) that, however, might represent promising antifungal targets [11]. To date, several metabolic routes fundamental for IPA manifestation have been identified: *de novo* UMP biosynthesis [12], the folate synthesis route [13], siderophore-mediated iron assimilation [14], or the methylcitrate cycle [15] are essential metabolic processes supporting *in vivo* growth and virulence of *A. fumigatus* [16]. Nevertheless, detailed knowledge about the metabolic status of the fungus during intrapulmonary growth is still scarce due to the complexity of the pathogen-host system. In a seminal study, preliminary insights into the *A. fumigatus*-host adaptation transcriptome were gained employing extensive transcriptional profiling under various culture conditions and during an early phase of pulmonary infection, revealing the main stressors encountered by the fungal pathogen when germinating in a susceptible, leukopenic mammalian host [17]. Among these, starvation for nitrogen became evident. Yet, while numerous studies have focused on *A. fumigatus* nitrogen metabolism [16], [18], [19], [20], [21], [22], neither the exact source(s) of this macroelement nor specific metabolic routes of nitrogen assimilation during pulmonary infection have been identified so far.

Sulphur (S) is another essential nutrient that the fungus needs to acquire from the surrounding tissue during intrapulmonary growth, as it is a constituent of the proteinogenic amino acids cysteine and methionine as well as of essential organic molecules like coenzyme-A, glutathione and, particularly, iron-sulphur (Fe-S) clusters. Only a few studies have addressed its relevance for fungal virulence so far, focusing on synthesis and utilization of the sulphur-containing molecule glutathione in *Candida albicans* or *C. glabrata* [23], [24]. The importance of sulphur metabolism for aspergillosis has not been addressed to date. *Aspergillus* species can utilize a variety of sulphur-containing molecules, such as inorganic S-sources, e.g. sulphate, or sulphate esters of organic compounds [25]. The assimilation of sulphate is performed *via* a well-defined pathway that comprises its uptake by specialised permeases, activation by ATP-driven phosphorylation in two steps, and reduction to sulphite and further to sulphide [26]. The latter is condensed with O-acetyl serine to yield cysteine, from which methionine and also S-adenosylmethionine can be formed. Sulphur homeostasis is tightly regulated in filamentous fungi by catabolite repression [27]. In the model filamentous fungus *Neurospora crassa* for instance, CYS-3, a positive-acting transcriptional factor of the bZIP family, was identified that activates expression of a set of genes whose products are required to acquire and utilize secondary S-sources under sulphur-starved conditions [28], [29], [30]. Furthermore, the role of so-called sulphur controller (*scon*) genes has been addressed that influence methionine repression of sulphur-containing amino acid biosynthesis [31]. Regulation of sulphur metabolism has also been extensively studied in the bakers' yeast *Saccharomyces cerevisiae* (for detailed information consult reviews [32] or [26]). While in *N. crassa* CYS-3 is sufficient to bind DNA and to activate transcription, in *S. cerevisiae* a heteromeric complex of three proteins (Met4-Met28-Cbf1) is required for proper sulphur-dependent regulation [33], [34], [35]. The CYS-3 orthologue of *A. nidulans*, MetR, is required to activate transcription of several genes related to sulphur acquisition and to allow growth on a variety of sulphur sources other than methionine [36]. Successful complementation of an *A. nidulans metRΔ* strain with its *MET1* orthologue from the dimorphic human-pathogenic fungus *Paracoccidioides brasiliensis* demonstrated functional conservation of this transcriptional regulator [37].

Adaptation to environmental stressors is usually based on reprogramming of the cellular expression pattern triggered by specific transcription factors. Consequently, the relevance of several cellular processes for *A. fumigatus* virulence has been investigated by targeting the corresponding regulators (for review see ref. [38]). Deletion of genes coding for zinc or iron responsive factors demonstrated the importance of both elements for virulence [39], [40]. Also, a positive role of amino acid homeostasis in *A. fumigatus* virulence was established by deletion of the respective cross-pathway control regulator [41]. A substantial benefit from targeting wide-domain regulators of a given cellular aspect lies in its comprehensive outcome, in contrast to particular gene deletions affecting activities that may be encoded redundantly in the fungal genome. Based on the hypothesis that flexible regulation of sulphur homeostasis supports growth in a susceptible host and therefore might affect virulence of *A. fumigatus*, we became interested in the cellular function of the MetR orthologue in this opportunistic pathogen. Our results demonstrate that this transcription factor is a key regulator of sulphur assimilation and that it is crucial for *A. fumigatus* pathogenicity. Moreover, we describe an unprecedented regulatory crosstalk of S-metabolism and iron homeostasis.

## Results

### The *Aspergillus fumigatus* genome encodes a highly conserved regulator of sulphur metabolism

**Figure 1:**
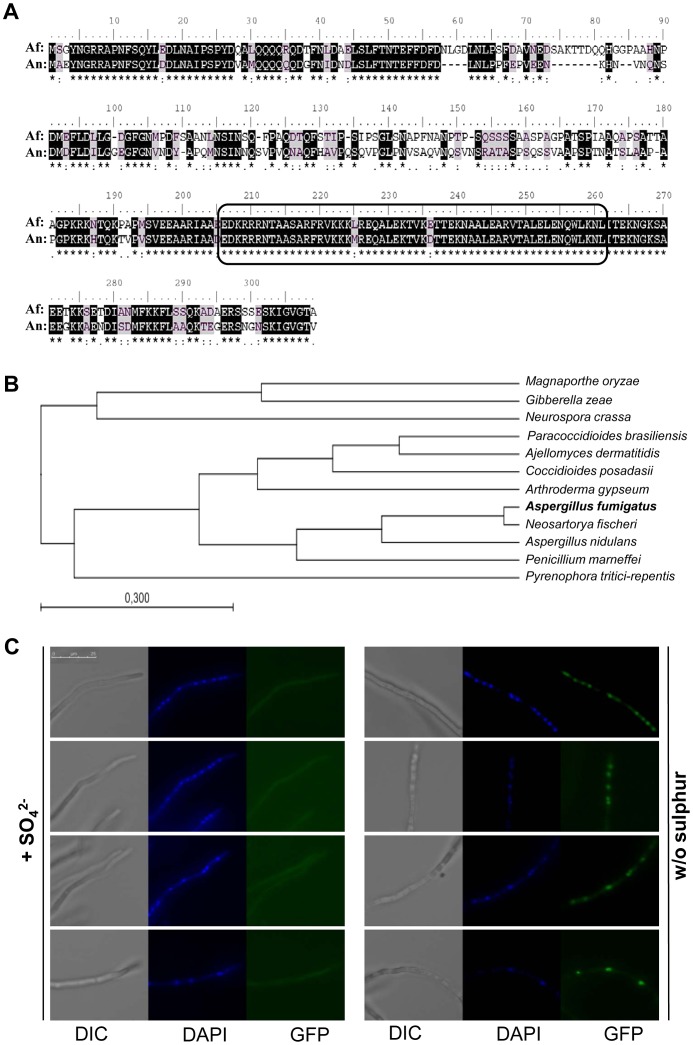
MetR of *Aspergillus fumigatus* is highly conserved and translocates to the nucleus upon sulphur starvation. (A) Alignment of *Aspergillus fumigatus* (Af) and *A. nidulans* (An) MetR proteins. Identical residues are shadowed in black and similar residues in grey. The bZIP region is framed in the black rectangle. (B) Exemplary molecular phylogenetic tree of the amino acid sequences of MetR/CYS-3 proteins of several ascomycota species. Multiple alignment and phylogenetic tree were created using CLC Sequence Viewer software (Workbench). Protein accession numbers are as follows: *Ajellomyces dermatitidis* EEQ78187, *A. fumigatus* EAL90135.1, *Arthroderma gypseum* EFQ98814, *A. nidulans* AF148535_1, *Coccidioides posadasii* EER26081, *Gibberella zeae* EAA74455.1, *Magnaporthe oryzae* EHA57141; *Neurospora crassa* AAA33585, *Neosartorya fischeri* EAW25471.1, *Paracoccidioides brasiliensis* EEH48461, *Penicillium marneffei* EEA27939, *Pyrenophora tritici-repentis* EDU47197. (C) Localisation of a functional MetR-GFP derivative in the presence or absence of sulphate as sole source of sulphur. Within 90 minutes of S-starvation, cytoplasmic localisation of the regulator changes to nuclear as demonstrated by co-localisation of the fluorescent signal with the nuclear stain DAPI.

The *A. fumigatus* MetR transcription factor was identified by BLAST search [42], [43], [44] on the NCBI server (http://www.ncbi.nlm.nih.gov/) using the *A. nidulans* MetR protein sequence (PubMed acc. no. AAD38380) as a query, revealing 64% identity and 75% similarity between both proteins (Fig. 1A). Notably, the leucine zipper (bZIP) domains of the factors are virtually identical with 97% identity and 100% similarity, which suggests that both could recognize a similar DNA target sequence.

In the C*A*DRE database [45] the *A. fumigatus metR* gene (AFUA_4G06530) had been automatically annotated to contain 1517 base pairs (bp), resulting in a predicted coding region of 918 nucleotides (nt) based on the presence of an unusually long intron 599 nt in size. The observable size difference in a 1% agarose gel between a *metR* cDNA (obtained by reverse transcription from mRNA) and gDNA (amplified from genomic DNA) matched with the presence of this intron (not shown), and sequencing of a complete cDNA insert confirmed the overall architecture of the *metR* gene as annotated. The deduced protein sequence comprises 305 amino acids with a predicted molecular weight of 33 kDa. The MetR transcription factor displays a high degree of conservation among ascomycetous fungi (Fig. 1B), being present in all genera analysed. With respect to the *A. fumigatus* MetR sequence, identities/similarities range from 25/33% to the *N. crassa* orthologue and up to 95/100% to the *Neosartorya fischeri* one. Also, the unusually long intron appears to be conserved among ascomycota, suggesting a common origin of the gene.

With the aim to probe localisation as well as any cellular function of the *metR* gene product, an *A. fumigatus* strain that would express a functional GFP-tagged version from the endogenous *metR* promoter was constructed. This strain AfS171 was shifted from sulphate-containing medium for 90 minutes to medium lacking any source of sulphur (Fig. 1C). Under S-rich conditions a faint fluorescent signal was observed that was uniformly distributed in the hyphal cytoplasm. Upon S-depletion, however, translocation of the MetR-GFP protein to the nuclei became evident, as deduced from co-localisation of the fluorescent signals with the nuclear stain 4′,6-diamidino-2-phenylindole (DAPI). Accordingly, the presumed DNA binding domain of MetR and its cytoplasmic-nuclear shuttling under sulphur-limiting conditions imply that this gene product acts as a transcriptional regulator of *A. fumigatus* sulphur metabolism.

### *A. fumigatus* requires MetR for assimilation of various sulphur sources

**Figure 2:**
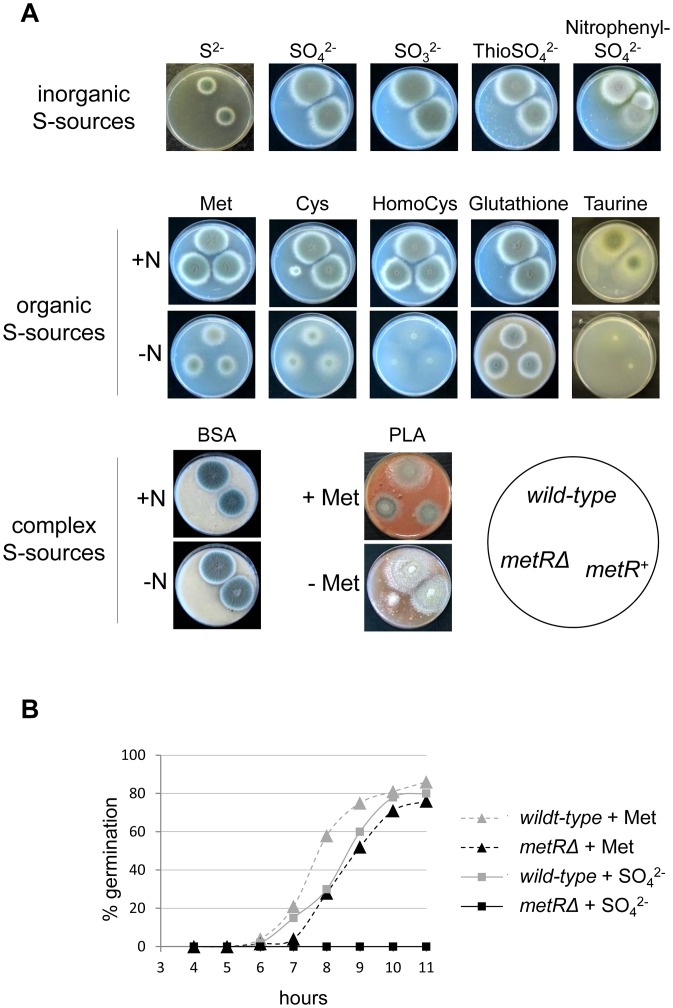
Phenotypic analysis and germination rate of an *A. fumigatus* strain deleted for its *metR* gene. (A) A *metRΔ* deletion mutant is unable to grow on any of the tested inorganic sulphur sources. Conidia of the indicated strains were inoculated on *Aspergillus* minimal medium supplemented with various sources of sulphur as well as complex media to monitor growth after three days of incubation at 37°C. Among the tested organic sources, the *metRΔ* strain grows only on methionine or homocysteine. When the media are depleted for nitrogen and sulphur simultaneously, cysteine and glutathione can be exploited as sources for both elements. The *metRΔ* strain also grows poorly on a substrate prepared from a porcine lung (PLA) unless supplemented with methionine. In all conditions, the phenotype of the *metR^+^* revertant strain was indistinguishable from the wild-type progenitor. (B) From conidia inoculated in liquid culture, rates of germination were deduced in dependency of the S-source. Germ tube formation of the *metRΔ* strain is slightly delayed with respect to the wild-type in the presence of 5 mM methionine as S-source, while in the presence of 2 mM sulphate, the mutant is not able to germinate within 11 hours of incubation.

To gain insights into the cellular function of the *metR* gene product, a full deletion strain of *A. fumigatus* was constructed by homologous gene replacement employing a self-excising recyclable cassette that contains a hygromycin B resistance gene as selectable marker [46], [47]. Southern analysis of the resulting strains AfS166 [*metR::six-β-rec/hygro^R^-six*] and AfS167 [*metR::six*] confirmed the homologous replacement and the excising event, respectively (Fig. S1A). A preliminary phenotypic analysis revealed that a *metR* deletant is unable to grow in the presence of sulphate as sole source of sulphur (Fig. 2A). This allowed us to reintroduce the *metR* gene at its original locus without using any selection marker but the presence of sulphate (SO_4_^2−^) as the only source of sulphur. In order to differentiate between the desired reconstituted strain and its wild-type progenitor, a silent mutation was introduced in the gene's coding sequence to create an additional *BstE*II restriction site. Southern analysis confirmed the correct integration event for a representative isolate (Fig. S1B).

To address the role of the MetR factor for the ability of *A. fumigatus* to utilize different sulphur sources, the resulting strains were subjected to phenotypic inspection on various S-containing media (Fig. 2A). With respect to inorganic S-sources, the *metR::six* (syn. *metRΔ*) mutant strain AfS167 was unable to grow in the presence of any of the tested substrates. Among the organic sources, the *metRΔ* mutant grew on methionine as well as homocysteine, which contrasts with a corresponding *A. nidulans metRΔ* mutant that was described to grow only poorly on the latter compound [32]. It was noticed, however, that homocysteine did not suffice as source of sulphur at alkaline pH while methionine was perfectly assimilated (not shown). Furthermore, AfS167 was able to utilize cysteine or glutathione as combined nitrogen and sulphur sources when the culture media were additionally depleted for nitrogen. Importantly, the *metRΔ* strain barely grew on porcine lung agar (PLA), suggesting that this mutant could suffer from a growth defect within the pulmonary tissue of a susceptible host and, therefore, might have reduced virulence capacities. When methionine was added to the PLA medium growth of the mutant was restored, which indicates that this tissue contains insufficient free levels of this amino acid to support growth of the deletant. In order to test for any cross-talk of utilisation pathways for other macro-elements, growth of the *metRΔ* mutant was monitored on various N-, C-, or P-sources in the presence of methionine and sulphate, respectively (Fig. S2). With one exception, no obvious phenotype emerged in dependency of the various supplements, i.e. the mutant was able to grow only when methionine was present as source of sulphur. However when galactose served as C-source, growth of the deletant appeared repressed even in the presence of methionine, which indicates that this particular sugar interferes with methionine utilisation in *A. fumigatus*.

To better understand the growth defect of AfS167 on inorganic S-sources, germination of the deletant was investigated (Fig. 2B). The strain was unable to swell or germinate in the presence of SO_4_^2−^ up to eleven hours after inoculation; on the contrary, its germination on methionine was only slightly delayed in comparison with the wild-type strain and reached approximately the same rate after prolonged incubation. Therefore, it appears that sulphur assimilation is an indispensable prerequisite for proper germ tube emergence and its absence results in a pronounced germination and growth defect as displayed by the *metR::six* mutant strain.

**Figure 3:**
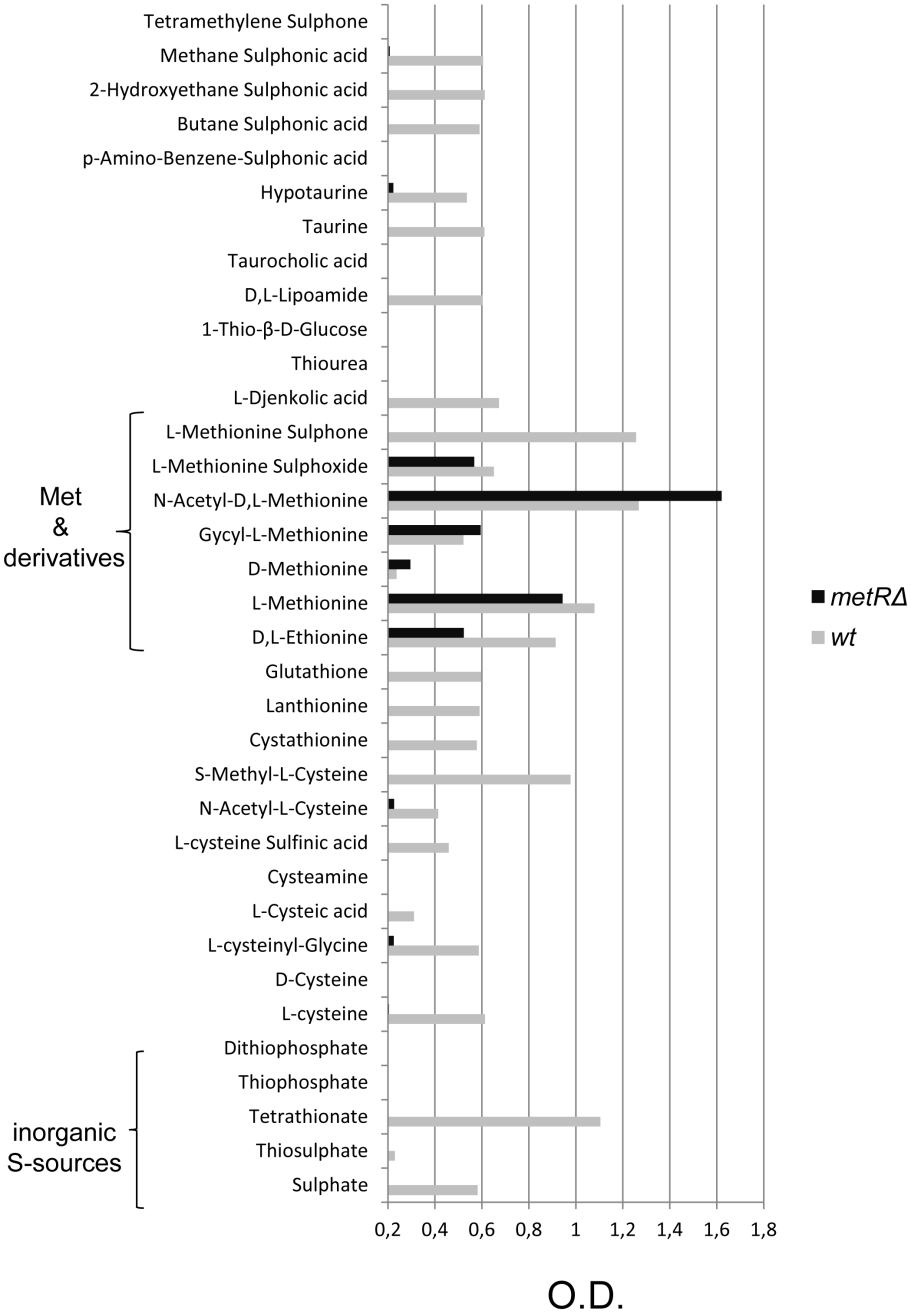
Phenotypic analysis by the Biolog microarray system. Wells of a Biolog PM 4 plate were inoculated with 100 µl aliquots of conidial suspensions prepared in sulphur-free minimal medium. Fungal growth was measured after 48 hours via the optical density (O.D.) at 630 nm, with an O.D. value of 0.2, calculated from the sulphur-depleted condition and confirmed through microscopic observation, serving as growth threshold. Out of the 35 different sulphur sources present in the Biolog PM4 plate, only methionine and derivatives thereof could be used as sulphur sources by the *metRΔ* strain. Importantly, none of the inorganic sulphur sources triggered growth of the mutant.

To further analyse the S-assimilation capacity of the deletion strain we made use of Biolog Phenotype MicroArrays (Fig. 3). This technology allows the evaluation of a variety of microbial phenotypes in a parallel fashion. From a variety of conditions and stressors available, the PM4 MicroPlate plate containing 35 different sulphur sources was selected. Wild-type and mutant strains were incubated in parallel in the wells of this microtiter plate and their growth ability in the presence of different S-sources was monitored *via* the optical density (O.D.) at 630 nm. The recommended Biolog Redox Dye to monitor growth by measuring the respiration process was also tested; however, as previously described for filamentous fungi [48], the results obtained using this dye turned out to be inconsistent (data not shown). Therefore, one well lacking any sulphur source was used to normalize O.D. values to define a growth threshold of 0.2, which was further confirmed through microscopic inspection. Since the use of phenotypic microarray plates is a technology that has not been extensively used for filamentous fungi yet, some of the compounds present in the PM4 MicroPlate were re-tested in regular phenotypic assays on solid culture media to support validity of the results (not shown). Growth phenotypes deduced from this Biolog plate confirmed that the *metRΔ* strain is unable to utilise any oxidized inorganic sulphur source. Indeed, it only grew on methionine or derivatives thereof (i.e. N-acetylmethionine or methionine sulphoxide). Notably, the mutant did not grow in the presence of methionine sulphone, although the wild-type strain grew perfectly well, but it grew better than its progenitor on N-acetylmethionine. The central conclusions reached from these phenotypic analyses are that the *metRΔ* mutant is unable to grow on inorganic sulphur sources and that utilization of methionine and its derivatives is independent from the presence of the transcriptional activator MetR.

### Utilisation but not production of volatile S-compounds by *A. fumigatus* depends on MetR

**Figure 4:**
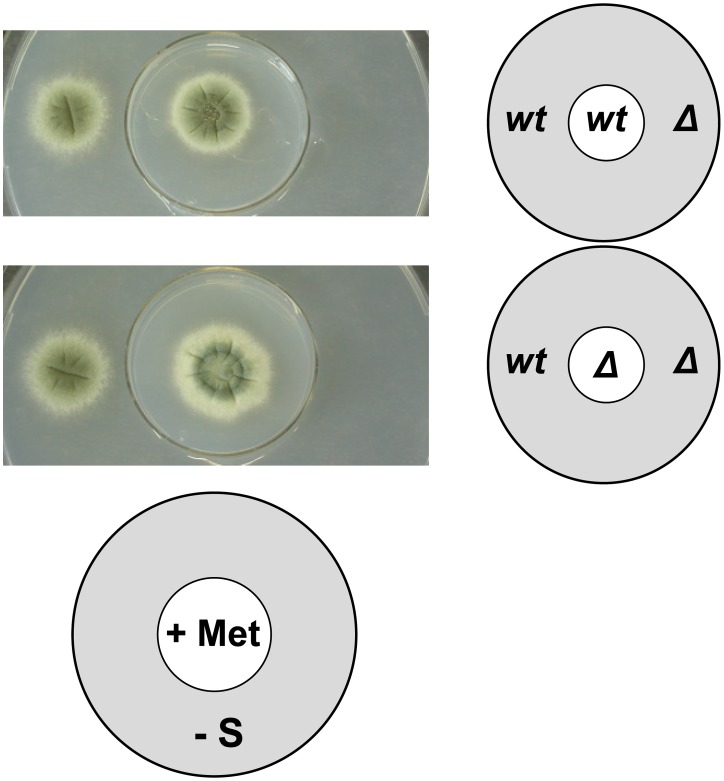
Utilization but not production of volatile sulphur compounds by *A. fumigatus* is independent of MetR. Cross-feeding experiments with the wild-type isolate and its *metRΔ* derivative were carried out on spatially separated culture media that allow gaseous exchange of volatile compounds. Growth of externally inoculated *A. fumigatus* demonstrates that this fungus can utilize volatile sulphur compounds (VSCs) that are produced in the course of methionine catabolism by the internally inoculated isolate. Production of VSCs does not require MetR, as demonstrated by growth of the wild-type isolate when the respective mutant strain is grown on methionine-containing medium. In contrast can the *metRΔ* strain not use VSC as sole source of sulphur.

Because the AfS167 strain is unable to grow in the presence of S^2−^ (Fig. 2A) and as it is known that several fungi, including *Aspergillus* species, produce volatile sulphur compounds (VSCs) like hydrogen sulphide (H_2_S), dimethylsulphide (H_3_C-S-CH_3_), or methanethiol (CH_3_-SH) as a result of methionine catabolism [49], [50], [51], we became interested in studying whether *A. fumigatus* would be able to utilize such volatile compounds as S-source and if generation of such VSCs is MetR-dependent. The wild-type and *metRΔ* strains were cultured in small petri dishes with minimal medium containing methionine as sulphur source. These plates were placed inside larger petri dishes with medium lacking any S-source. Neither the wild-type isolate nor the mutant was able to grow in the absence of any sulphur source (not shown). However, when either strain was grown on the methionine-containing petri dish, growth on the outside sulphur-depleted medium was observed only for the wild-type strain (Fig. 4). Accordingly, *A. fumigatus* is able to take up VSCs produced from methionine catabolism and to use them as S-source. Production of VSCs appears to be independent from the presence of the MetR regulator but their utilization as S-source requires the presence of this regulatory factor.

In the light of these results, it is valid to conclude that the MetR factor represents a master regulator of sulphur assimilation in *A. fumigatus* acting predominantly on utilization of inorganic S-sources, while consumption of methionine does not depend on this transcriptional regulator.

### MetR mediates transcriptional activation of sulphur assimilation genes

The aforementioned phenotypic results suggest that the MetR factor might activate the transcription of genes required for the uptake and utilization of different sulphur sources. To investigate this, the short-term transcriptional responses of a wild-type strain and its *metRΔ* derivative to variations in the available sulphur source were monitored: Mycelia grown overnight in *Aspergillus* minimal medium (AMM) with methionine were shifted to media containing diverse S-sources, incubated for one additional hour, and steady-state levels of several transcripts of genes related to sulphur uptake and utilization were monitored by Northern blot hybridisation.

**Figure 5:**
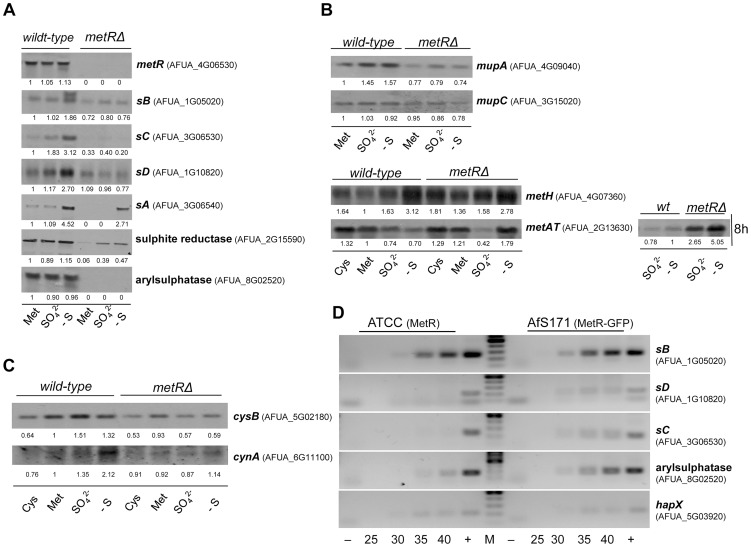
Transcription analysis of sulphur-related genes in the presence of varying sulphur sources. Analysis of the transcriptional expression of several genes that participate in sulphur metabolism by Northern blot hybridisation. RNA samples had been isolated from the *metRΔ* deletant and its wild-type progenitor strain after shifting pre-grown fungal cultures to medium containing the indicated source of sulphur (Met, Cys, and SO_4_^2−^) or lacking any S-Source (-S). Transcript steady-state levels were monitored from cultures after one hour of growth with the exception of the *metAT* transcript, for which culturing had been extended to eight hours additionally. For all blots, rRNAs served as loading control, autoradiographies are representatives from three independent, reproducible experimental replicates. (A) Expression of the *metR* gene itself is not regulated by the sulphur source. Expression of all genes from the sulphate assimilation pathway - *sA*, *sB*, *sC*, and *sD* - is elevated under sulphur starving conditions in a MetR-dependent manner. Expression of the arylsulphatase encoding gene completely depends on MetR. (B) Expression of the putative methionine transporter encoding genes (*mupA* and *mupC*) is constitutive. Transcription of the methionine synthase encoding gene (*metH*) is upregulated under sulphur-starving conditions, independently of MetR. Regulation of the expression of the methionine aminotransferase encoding gene (*metAT*) is MetR-independent. Expression of *metAT* is elevated in the presence of methionine. In addition, the *metRΔ* mutant increases *metAT* expression under sulphur-depleted conditions. This upregulation also occurred after eight hours incubation in the presence of sulphate. (C) Transcription of the cysteine synthase (*cysB*) is slightly upregulated with a sulphur source other than cysteine in a MetR dependent manner. Expression of the putative cysteine permease (*cynA*) is upregulated under sulphur-starving conditions in a MetR-dependent manner. (D) MetR binds to the promoter regions of selected candidate genes of the sulphate assimilation pathway, like *sB*, *sD*, *sC*, or AFUA_8G02520 but not the iron regulator gene *hapX* as demonstrated by chromatin immunoprecipitation analyses. Shown are inverse images from agarose gel electrophoreses after semi-quantitative PCRs on fixed and sheared chromatin samples enriched from the MetR-GFP strain AfS171 in comparison to samples from the untagged wild-type control strain ATCC 46645; − and + specify negative (without template) and positive (genomic DNA as template) controls, respectively, while figures indicate the numbers of PCR cycles and M stands for the DNA size standard.

Initially, transcript levels of the *metR* gene itself were checked to observe that its transcription is apparently not regulated by the nature of the sulphur source (Fig. 5A), resembling the situation in *A. nidulans* [32] but contrasting findings in *N. crassa* [24], [25]. Interestingly, a second hybridising signal was detected for the *metR* transcript under S-starvation conditions, indicating alternative processing of the encoding transcript. In order to further understand why the *metRΔ* mutant is unable to grow on oxidized inorganic sulphur sources, expression of all genes encoding enzymes of the sulphate assimilation pathway, which are sulphate permease (*sB*), ATP-sulphurylase (*sC*), APS-kinase (*sD*), PAPS-reductase (*sA*) and sulphite reductase, as well as expression of one arylsulphatase-encoding gene (required for utilization of sulphur esters, i.e. nitrophenyl sulphate) was checked (Fig. 5A). Upregulation of the sulphate permease-, ATP-sulphurylase- and APS-kinase-encoding genes under sulphur-starving conditions depended on the presence of MetR factor. For the *sB* gene, a second, longer transcript became evident under sulphur starvation. Transcription levels of the genes coding for PAPS reductase and sulphite reductase were decreased in the absence of the MetR factor on all S-sources in comparison to the wild-type. Furthermore, expression of the arylsulphatase-encoding gene was completely shut down in the mutant. This transcriptional pattern agrees with and partially explains the incapacity of the *metRΔ* deletant to grow on inorganic sulphur sources.

In contrast to inorganic sulphur sources, the AfS167 deletion mutant grows on methionine independently of other conditions such as varying pH (not shown) or the availability of nitrogen. To address the reason for this phenotype, transcription of several methionine-related genes was investigated (Fig. 5B). A BLAST search against the *A. fumigatus* genome sequence identified three putative transporters involved in methionine uptake, which we named *mup* genes. Transcript levels of *mupA* and *mupC* were constitutive with respect to the sulphur source and seemed to be mostly independent of MetR, although *mupA* expression appeared somewhat elevated in the wild-type. Transcription of the related *mupB* gene could not be detected under any condition tested (not shown). Transcription of the putative methionine synthase-encoding gene *metH* was also not regulated by MetR. Interestingly, its expression was reduced in the presence of methionine and increased under sulphur-starving conditions, suggesting that the intracellular pool of methionine is constantly maintained and emphasizing the importance of this particular amino acid. Importantly, the methionine aminotransferase-encoding gene *metAT*, whose product is probably responsible for methionine degradation [52], [53] and consequently for its utilization as sulphur source, was highly expressed in the presence of methionine in a MetR-independent manner. Therefore, the expression pattern of these genes perfectly agrees with the ability of the deletion strain to utilize methionine as S-source. Interestingly in the *metRΔ* mutant, *metAT* was highly expressed under sulphur-depleted conditions. We hypothesized that this is due to the strong and rapid sulphur starvation affecting the mutant under such sulphur-restricted conditions. To verify this assumption, the wild-type and mutant strains were incubated up to eight hours on sulphate-containing and sulphur-depleted media (Fig. 5B), which results in substantial sulphur-limiting conditions for the deletant but not for the wild-type. Accordingly, *metAT* expression was upregulated in the *metRΔ* mutant but not in the wild-type, demonstrating that after prolonged incubation in the presence of sulphate the mutant strain becomes depleted for sulphur. Surprisingly, the wild-type did not upregulate expression of *metAT* even after eight hours of incubation under sulphur-depleted conditions, implying that the resulting sulphur starvation in the wild-type is not that severe, probably due to MetR-dependent recycling processes and mobilization of reserves. In conclusion, the *metAT* expression profile suggests that other mechanisms apart from MetR-mediated regulation must exist to orchestrate gene expression depending on the availability and source of sulphur.

With respect to cysteine assimilation no clear candidates for its degradation and utilization as sulphur source have been identified so far and, consequently, the incapacity of the deletion strain AfS167 to grow on cysteine could not be addressed properly. Nevertheless, expression of the putative cysteine transporter-encoding gene *cynA*, an orthologue to a *C. glabrata*-specific cysteine transporter [54], was not upregulated in the *metRΔ* strain under sulphur starving conditions (Fig. 5C), which would partially explain the observed phenotype. Furthermore, transcription of the cysteine synthase-encoding gene *cysB* was slightly increased in the absence of cysteine in a MetR-dependent manner, which might translate into a slightly reduced level of cysteine in the deletion strain.

To demonstrate a direct effect of MetR on the transcription of sulphur assimilation genes, we performed chromatin immuneprecipitation (ChIP) analyses making use of strain AfS171 expressing a functional GFP-tagged version of this transcription factor. Fixed chromatin samples isolated from fungal cultures that had been starved for sulphur were sheared and precipitated with a nano-trap (see *Supporting Information* for details). Interrogating the output fractions by semi-quantitative PCR revealed a pronounced and reproducible enrichment of fragments spanning the promoters of several candidate genes, such as the ones encoding the APS-kinase, the arylsulphatase, the ATP-sulphurylase, as well as the sulphate permease (Fig. 5D).

### MetR is required for broad transcriptional remodeling upon sulphur deprivation

**Figure 6:**
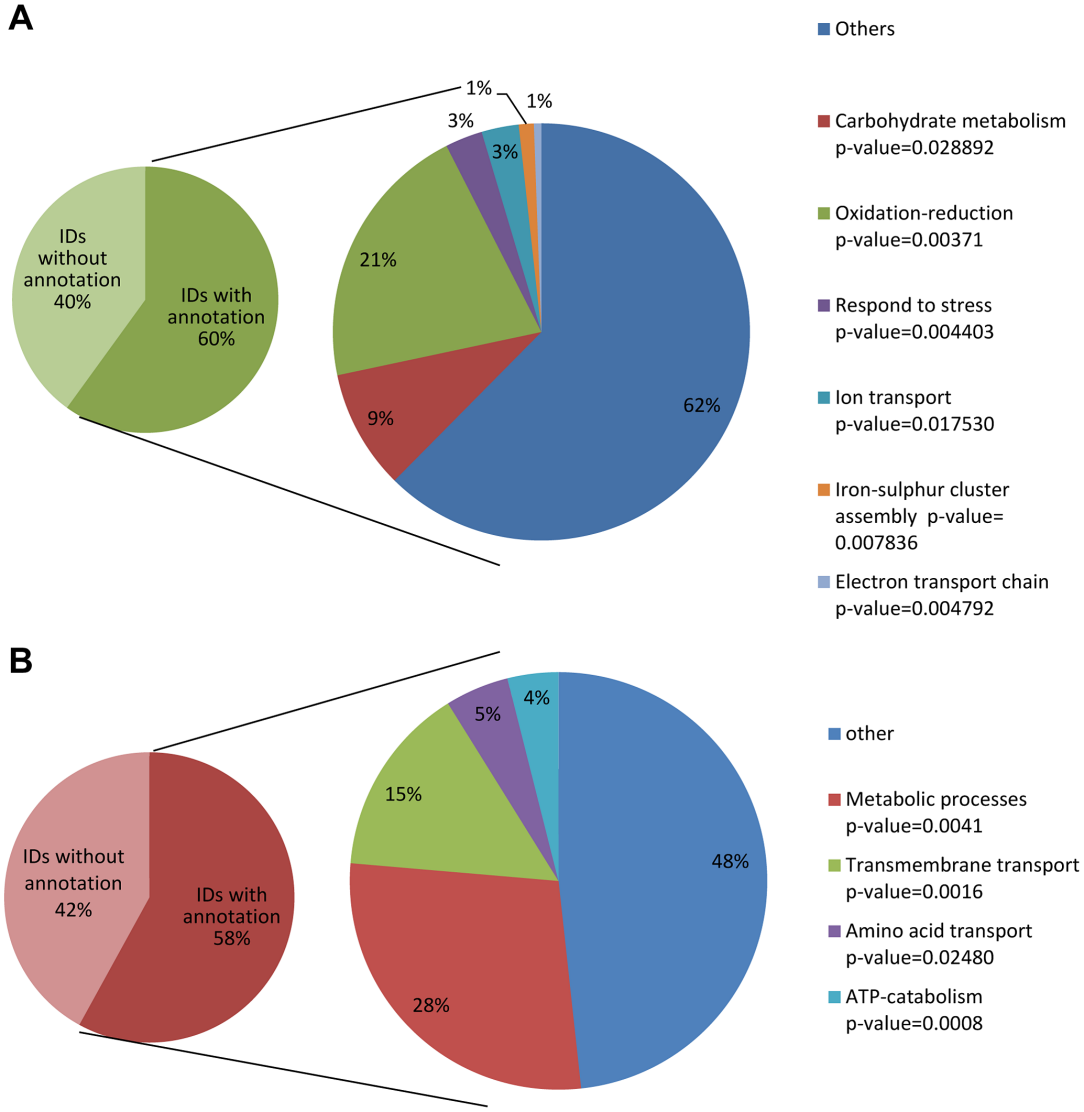
Functional categories of genes regulated by MetR as deduced from RNA-seq data. Categorisation was performed *via* the FungiFun suite (https://sbi.hki-jena.de/FungiFun/FungiFun.cgi) to identify functional groups for genes that are less (A) and more abundantly (B) expressed in the *metRΔ* mutant AfS167 compared to its wild-type progenitor ATCC 46645 after eight hours of culture under sulphur-limiting conditions. The minor pie charts (left panels) indicate the percentage of genes with and without annotation in the GO database; the larger pie charts (right panels) represent the deduced functional groups of the annotated genes and indicate percentages of genes for each category.

Following the observation that the expression of genes whose products are required for sulphur assimilation is regulated by MetR, we became interested in understanding to what extent any transcriptional remodeling that takes place under sulphur-limiting conditions is MetR-dependent. For this purpose, overnight-grown mycelia of the wild-type and *metRΔ* strains were shifted from cultures containing sufficient levels (5 mM) of methionine serving as sole S-source to media containing low methionine levels (0.2 mM) over a time frame of eight hours before RNA was harvested. Previous culturing experiments had shown that methionine depletion became manifest within this time frame, so this experimental set-up allows assessment of any MetR contribution to the transcriptional response upon mild S-depletion. Nucleic acid samples were prepared from two biological replicates each to perform digital transcriptome analyses by the RNA-seq approach (see *Materials and Methods* for details). Comparison of both transcriptomes under this specific condition revealed that 288 genes were downregulated and 349 were upregulated in the *metRΔ* strain with respect to its wild-type progenitor (>1.5-fold change, p-value<0.05) (Table 1 and Table S1). Categorisation *via* the FungiFun suite [55] revealed that the main cellular functions affected by the absence of MetR are membrane transport, metabolism, carbohydrate metabolism, and oxidation/reduction (Fig. 6). Therefore, MetR action is required for the correct remodeling of these processes to counteract conditions of sulphur depletion. To further understand this adaptation, we performed a deeper functional categorisation. Various genes assigned to cation homeostasis were less abundant in the *metRΔ* mutant (Table 2), suggesting a strong dysfunction in the regulation of the metabolism of these ions. In addition, several genes whose products participate in sugar, glucoside, polyol and carboxylate metabolism were downregulated what highlights the greatly different metabolic status of the mutant under sulphur starvation. Furthermore, genes related to cellular export and secretion were also identified, hinting a distinct interaction with the environment. Surprisingly, several genes related to mRNA synthesis and were also found to be downregulated, which indicates a interplay of MetR regulation with other transcription factors and cell cycle regulation.

**Table 1:** Top 20 genes with lower and higher expression in AfS167.

Genes with lower expression in the *metRΔ* mutant compared to wild-type			
Gene ID	Description	fold change	p-value
AFUA_2G17940	MAK1-like monooxygenase, putative	0.090	3,9E-22
AFUA_8G00300	conserved hypothetical protein	0.098	0,00292
AFUA_5G00410	conserved hypothetical protein	0.103	2,7E-19
AFUA_5G00730	H/K ATPase α subunit, putative	0.129	2,4E-48
AFUA_4G04318	copper resistance protein Crd2, putative	0.165	3,4E-37
AFUA_5G10210	conserved hypothetical protein	0.172	7,4E-05
AFUA_4G00460	chlorohydrolase family protein, putative	0.173	9,1E-10
AFUA_4G00450	hypothetical protein	0.175	2,9E-09
AFUA_3G13640	extracellular serine-rich protein, putative	0.193	0,00108
AFUA_7G01180	extracellular lipase, putative	0.205	4,5E-08
AFUA_8G01870	hypothetical protein	0.207	3,2E-07
AFUA_8G01980	conserved hypothetical protein	0.213	4,5E-28
AFUA_8G01530	HHE domain protein	0.214	2,9E-05
AFUA_8G01520	pectin methylesterase, putative	0.218	1,9E-05
AFUA_2G12680	conserved hypothetical protein	0.222	2,9E-11
AFUA_3G12910	O-methyltransferase GliM-like, putative	0.231	2,1E-24
AFUA_8G01970	extracellular endo-polygalacturonase, putative	0.232	2,5E-25
AFUA_3G12900	MFS transporter, putative	0.233	3,8E-24
AFUA_8G06510	conserved hypothetical protein	0.238	1,8E-05
AFUA_2G04200	probable 4-hydroxyphenylpyruvate dioxygenase 1	0.239	1,1E-06

Genes with higher expression in the *metRΔ* mutant compared to the wild-type			
Gene ID	Description	fold change	p-value
AFUA_3G03420*	fusarinine C NRPS SidD (SM)†	65.45	1,2E-14
AFUA_3G03400*	siderophore biosynthesis acyltransferase SidF (SM)†	59.40	6,5E-09
AFUA_3G03430*	ABC multidrug transporter SitT	53.97	2,4E-12
AFUA_3G03410*	enoyl-CoA hydratase/isomerase SidH (SM)†	35.45	1,2E-10
AFUA_7G06140	β-*D*-glucoside glucohydrolase	26.38	2,2E-17
AFUA_7G06130	plasma membrane protein Pth11-like, putative	23.16	3,1E-27
AFUA_3G03380	sugar O-acetyltransferase, putative	17,14	3,6E-50
AFUA_3G03390*	siderophore biosynthesis lipase/esterase	14.89	4,6E-50
AFUA_2G07680*	*L*-ornithine *N^5^*-oxygenase SidA (SM)†	14.22	2,2E-74
AFUA_3G07640	plasma membrane H^+^-ATPase	14.01	2,2E-74
AFUA_3G03440*	MFS siderophore iron transporter (SM)	13.99	3,4E-12
AFUA_3G03445*	short-chain alcohol dehydrogenase, putative	13.65	1,2E-11
AFUA_7G07030	vegetative incompatibility WD repeat protein, putative	13.42	0,01565
AFUA_1G17190*	long-chain-fatty-acid-CoA ligase SidI (SM)†	12.70	0,01015
AFUA_8G06100*	integral membrane protein	12.19	0,00010
AFUA_8G06090	amino acid permease, putative	12.17	0,00015
AFUA_3G03450	cytochrome P450 oxidoreductase, putative	10.83	1,3E-14
AFUA_3G00710	similar to allergen Asp f4	10.32	2,0E-14
AFUA_7G04730*	siderochrome iron transporter, putative	8.90	0,00017
AFUA_8G06132	FluG domain protein	8.49	2,8E-11
AFUA_4G01510	C6 transcription factor, putative	7.23	0,02579

* SreA target genes [57];

† genes with proven function [56];

SM: siderophore metabolism.

**Table 2:** Categorization of genes with lower expression under S-starvation in the *metRΔ* mutant.

Gene ID	description	fold change	p-value
**Homeostasis of cations** (p-value = 0.00018)			
AFUA_5G00730	H/K ATPase α subunit, putative	0.129	2,4E-48
AFUA_4G04318	copper resistance protein Crd2, putative	0.165	3,4E-37
AFUA_2G17530	conidial pigment biosynthesis oxidase Arb2	0.260	0,00149
AFUA_4G10690	iron-sulfur cluster assembly accessory protein Isa1	0.381	3,7E-07
AFUA_7G04550	serine/threonine protein kinase, putative	0.428	4,7E-11
AFUA_7G04570	Na/K ATPase α 1 subunit, putative	0.443	2,4E-10
AFUA_8G01560	aldo-keto reductase (YakC), putative	0.458	0,00081
AFUA_5G11260	siderophore transcription factor SreA	0.505	3,0E-06
AFUA_1G15970	aldo-keto reductase (AKR13), putative	0.510	0,00262
AFUA_1G04680	NifU-related protein	0.534	0,00080
AFUA_4G11240	α-aminoadipate reductase large subunit, putative	0.545	0,00090
AFUA_4G12530	vacuolar iron importer CccA	0.552	2,0E-05
AFUA_3G09970	vacuolar iron importer, putative	0.558	0,00150
AFUA_2G10690	MFS phosphate transporter, putative	0.576	0,00062
AFUA_4G13540	potassium uptake transporter, putative	0.579	7,1E-05
AFUA_6G11300	integral membrane channel protein, putative	0.593	0,00157
AFUA_4G04150	di-, tri-valent inorganic cation transporter, putative	0.599	0,00021
AFUA_2G03860	membrane zinc transporter ZrfB	0.665	0,00126
**Sugar, glucoside, polyol and carboxylate metabolism** (p-value = 0.0052)			
AFUA_8G01970	extracellular endo-polygalacturonase, putative	0.232	2,5E-25
AFUA_2G14150	endo-arabinanase, putative	0.320	1,1E-13
AFUA_3G00470	endo-1,4-β-xylanase, putative	0.364	8,0E-05
AFUA_3G14270	aldo-keto reductase (AKR), putative	0.435	2,0E-10
AFUA_3G14620	extracellular endo-1,5-α-*L*-arabinase, putative	0.435	9,8E-05
AFUA_5G10370	succinate dehydrogenase iron-sulphur protein	0.449	7,4E-07
AFUA_8G07030	endo-β-mannanase fragment	0.467	0,00109
AFUA_2G10240	NAD binding Rossmann fold oxidoreductase, putative	0.497	1,9E-05
AFUA_2G10230	inositol oxygenase, putative	0.506	9,7E-05
AFUA_2G11240	UDP-N-acetyl-glucosamine-1-P transferase Alg7, putative	0.561	0,00018
AFUA_2G14520	hydrolase, putative,	0563	3,3E-05
AFUA_3G07810	succinate dehydrogenase subunit Sdh1, putative	0.582	1,5E-05
AFUA_1G06810	aconitate hydratase, mitochondrial	0.597	7,4E-05
AFUA_5G09680	succinate dehydrogenase cytochrome b560 subunit	0.602	0,00223
AFUA_4G13530	α,α-trehalase TreB/Nth1	0.652	0,00177
AFUA_4G11280	GPI mannosyltransferase 2	0.663	0,00211
**Cellular export and secretion** (p-value = 0.0087)			
AFUA_4G01140	MFS multidrug transporter, putative	0.331	9,1E-09
AFUA_8G06554	carbonate dehydratase, putative	0.336	0,00149
AFUA_8G06550	carbonic anhydrase family protein	0.347	0.00128
AFUA_5G02700	MFS multidrug transporter, putative	0.397	1,2E-11
AFUA_1G16910	MFS multidrug transporter, putative	0.440	0,00049
AFUA_5G08150	ABC bile acid transporter, putative	0.481	1,7E-07
AFUA_3G03700	MFS sugar transporter, putative	0.549	0,00064
AFUA_5G00420	MFS transporter, putative	0.562	0,00160
AFUA_1G15490	MFS multidrug transporter, putative	0.574	0,00122
AFUA_3G14560	MFS multidrug transporter, putative	0.589	0,00285
AFUA_2G04080	GPR/FUN34 family protein	0,604	0,00013
AFUA_4G11250	carbonic anhydraseCarbonic anhydrase Nce103, putative	0.641	0,00047
AFUA_5G07020	ribosome biogenesis ABC transporter Arb1, putative	0.652	0,00088
**mRNA synthesis** (p-value = 0.0113)			
AFUA_4G00950	mediator of RNA polymerase II transcription subunit 17	0.256	0,00023
AFUA_1G04110	C2H2 transcription factor, putative	0.410	3,2E-08
AFUA_7G04580	TBC domain protein, putative	0.451	5,9E-10
AFUA_2G07710	mRNA splicing factor RNA helicase	0.458	3,4E-09
AFUA_2G02520	cell polarity protein (Tea1), putative	0.465	8,2E-08
AFUA_4G07280	cAMP-mediated signaling protein Sok1, putative	0.503	5,5E-07
AFUA_3G10830	glutathione S-transferase GstA	0.503	2,9E-05
AFUA_5G11260	siderophore transcription factor SreA	0.505	3,0E-06
AFUA_6G03510	flavin containing polyamine oxidase, putative	0.515	1,6E-05
AFUA_5G08160	cyclin, putative	0.536	7,2E-06
AFUA_6G13340	mismatch-specific thymine-DNA glycosylase, putative	0.630	0,00246

Among the transcripts that are more abundant in the mutant (Table 3), several genes whose products participate in amino acid transport were of special interest as this implies a link between nitrogen and sulphur assimilation. In addition, various genes related to DNA conformation and repair were found to be upregulated, which might reflect the severe stress situation for the mutant under sulphur starving conditions. Intriguingly, several genes related to cation transport, and especially siderophore transport and reductive iron assimilation, were found to be upregulated, suggesting a connection of MetR-mediated regulation to iron homeostasis. Indeed, among the 20 genes that showed a higher expression in the mutant compared to the wild-type (Table 1), 11 genes that have previously been found to be upregulated during iron starvation dependent on the iron regulator SreA were identified, including five genes of proven function in siderophore biosynthesis [56], [57]. Further inspection of the entire list of upregulated genes revealed 29 of the known 49 SreA target genes with 13 of proven function in siderophore metabolism, reductive iron assimilation and iron regulation (not shown).

**Table 3:** Categorization of genes with higher expression under S-starvation in the *metRΔ* mutant.

Gene ID	description	fold change	p-value
**Homeostasis of metal ions** (p-value = 0.0016)			
AFUA_8G06920	K^+^ homeostasis protein Kha1, putative	1.562	0,00281
AFUA_2G15130	ABC multidrug transporter, putative	1.569	0,00294
AFUA_5G02290	potassium ion transporter (Trk1), putative	1.596	0,00078
AFUA_4G09560	Membrane zinc transporter ZrfC	1.806	0,00296
AFUA_4G06570	Ras guanine-nucleotide exchange protein, putative	2.097	4,7E-07
AFUA_3G12740	copper resistance-associated P-type ATPase, putative	2.127	1,1E-08
AFUA_2G05330	vacuolar H^+^/Ca^2+^ exchanger	2,999	0,04960
AFUA_3G07640	plasma membrane H^+^-ATPase	14.014	4,4E-78
**Iron acquisition: siderophore metabolism (SM) and red. iron assim. (RIA)** (p-value = 0.0068)			
AFUA_3G02980	metalloreductase Fre8 (RIA)	2.100	5,2E-05
AFUA_7G06060	MFS siderophore iron transporter Sit1 (SM)	3.002	5,1E-17
AFUA_3G03640	MFS siderophore iron transporter MirB (SM)	3.616	0,02636
AFUA_1G17270	metalloreductase FreB (RIA)	4.367	0,00010
AFUA_5G03800	high-affinity iron permease FtrA(RIA)	4.430	9,1E-14
AFUA_5G03790	ferrooxidoreductase Fet3 (RIA)	4.544	1,4E-12
AFUA_7G04730	MFS siderophore iron transporter (SM)	8.906	3,1E-06
AFUA_3G03440	MFS siderophore iron transporter (SM)	13.994	1,2E-14
AFUA_3G03400	siderophore biosynthesis acyltransferase SidF (SM)†	59.405	6,5E-09
AFUA_3G03420	fusarinine C NRPS SidD (SM)	65.458	3,2E-17
**DNA processing** (p-value = 0.043)			
AFUA_5G11700	DNA mismatch repair protein Mlh1, putative	1.574	0,00326
AFUA_3G08520	SRF-type transcription factor RlmA	1.642	0,00124
AFUA_7G05270	COMPASS complex subunit Sdc1, putative	1.656	0,00128
AFUA_4G11480	C2H2 finger domain protein, putative	1.758	0,00010
AFUA_4G11140	DNA polymerase iota, putative	1.854	0,00018
AFUA_3G12750	crossover junction endonuclease mus81	2.090	2,3E-08
AFUA_5G11170	nucleosome remodeling complex ATPase subunit	2.169	8,6E-08
AFUA_8G01090	thioredoxin, putative	2.241	0,00037
AFUA_1G02270	ARS binding protein Abp2, putative	2.696	2,0E-07
AFUA_1G15550	homeobox and C2H2 transcription factor, putative	2.697	1,7E-10
**Resistance proteins and transporters** (p-value = 0.00036)			
AFUA_2G15130	ABC multidrug transporter, putative	1.569	0,00294
AFUA_1G10370	MFS multidrug transporter, putative	1.620	0,00121
AFUA_3G08530	MFS drug transporter, putative	1.859	4,5E-05
AFUA_1G10390	ABC multidrug transporter, putative	1.959	0,00030
AFUA_4G14300	dynamin family GTPase, putative,	2.071	0,00315
AFUA_2G11420	MFS transporter, putative	2.405	0,00268
AFUA_7G00480	ABC multidrug transporter, putative	2.527	0,00245
AFUA_6G11890	dynamin GTPase, putative	3.601	3,9E-10
AFUA_6G01900	flavin-binding monooxygenase-like protein	4.245	3,3E-18
AFUA_4G14130	ABC multidrug transporter, putative	4.509	0,00056
AFUA_1G12690	ABC multidrug transporter Mdr4	6.200	7,6E-08
AFUA_1G03200	MFS transporter, putative	6.895	0,00120
AFUA_3G03430	ABC multidrug transporter SitT	53.978	8,6E-15
**Non-ribosomal peptide synthesis** (p-value = 0.02369)			
AFUA_1G17200	Nonribosomal peptide synthetase Fragment	1.894	2,7E-06
AFUA_1G10380	Putative non-ribosomal peptide synthetase Fragment	1.921	5,1E-06
AFUA_3G03420	fusarinine C NRPS SidD	65.458	3,2E-17
**Amino acid transport** (p-value = 0.0378)			
AFUA_7G01090	proline permease PrnB	1.813	0,00340
AFUA_1G12310	GABA permease	2.170	0,00159
AFUA_7G00440	GABA permease, putative	2.589	0,00031
AFUA_4G10090	GABA permease, putative	2.624	0,00152
AFUA_8G02760	mitochondrial ornithine carrier protein (AmcA), putative	2.762	0,00056
AFUA_5G00710	GABA permease, putative	2.938	3,1E-05
AFUA_8G06090	amino acid permease, putative	12.176	2,7E-06

In conclusion, the MetR-dependent adaptation to sulphur starvation conditions is a complex process that involves broad transcriptional remodeling to achieve altered expression of genes belonging to various functional categories.

### MetR is important for *A. fumigatus* virulence

In order to address the role of fungal sulphur utilization for growth and therefore virulence in a susceptible host, the involvement of the sulphur-related transcriptional regulator MetR in *A. fumigatus* virulence was assessed in different animal models. Initially, the alternative host model of the wax moth larvae *Galleria mellonella* was assayed (Fig. S3), where the *metRΔ* mutant displayed a significantly reduced virulence (p-value of <0.001) similar to the reduction observed for the control strain, an avirulent *pabaAΔ* mutant. This decrease in virulence was specifically attributed to the absence of *metR*, since the reconstituted strain recovered full virulence. Interestingly, when injected in a solution containing 5 mM methionine, the *metRΔ* strain was able to kill larvae as the wild-type, suggesting that the decrease in virulence is due to the absence of a proper source of sulphur.

**Figure 7:**
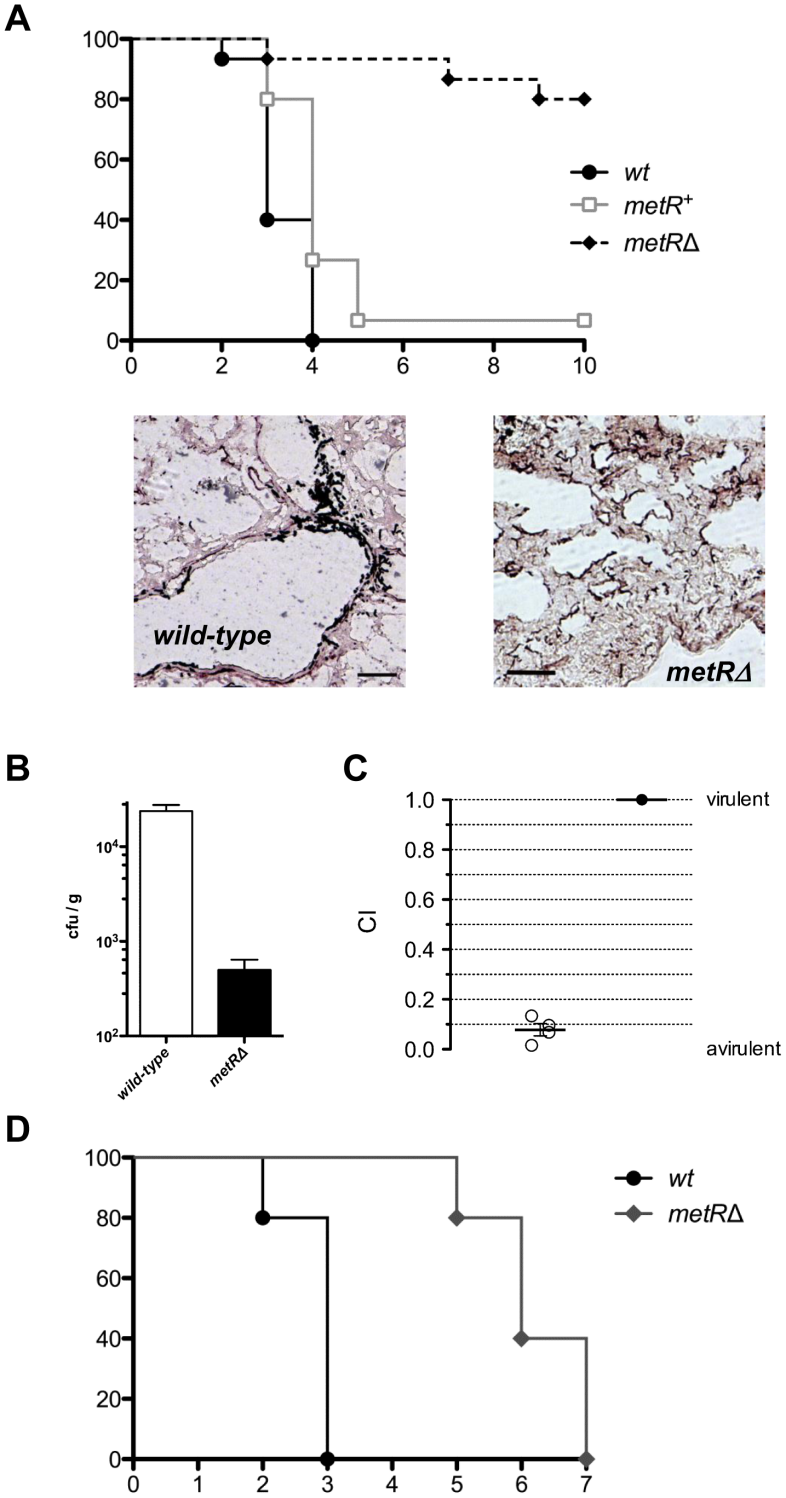
Virulence analysis of the *metRΔ* deletion strain. (A) The *metRΔ* mutant displays a significant (p<0.05) reduction in virulence when tested in a pulmonary infection model of leukopenic mice (n = 12–15 animals per group), with more than 80% of the infected animals surviving while full virulence was restored in the reconstituted strain. The representative histopathology sections demonstrate invasive growth for the wild-type strain, while any fungal structures had been cleared in animals infected with conidia of the *metRΔ* strain (scale bar equals 100 µm) (B) Fungal burdens determined by quantifying colony forming units on Met-supplemented culture medium from homogenised pulmonary tissues of mice (n = 5 per group) infected with the wild-type isolate or its *metRΔ* derivative. (C) In competitive infection experiments, susceptible animals (n = 4) were intranasally infected with equal amounts of conidia from wild-type and the *metRΔ* strain and sacrificed after four days. Aliquots from homogenized pulmonary tissues were then spread onto media containing or lacking methionine as sulphur source to differentiate between the *metRΔ* deletant and its wild-type progenitor. The resulting numbers of colony forming units were used to calculate the ratio between both strains before and after the infections. A mean competitive index (CI) of less than 0.1 can be deduced, illustrating that more than 90% of the recovered fungus represents wild-type and that the *metRΔ* deletant is virtually avirulent. (D) In a systemic murine infection model with leukopenic mice (n = 5 mice per cohort) infected intravenously with conidia from the *metRΔ* strain a significantly delayed disease progression in comparison to infections with the wild-type isolate became evident.

The results obtained in the wax moth model encouraged us to perform infections in established mouse models of aspergillosis. When challenging immunosuppressed, leukopenic mice intranasally with conidia of the *metRΔ* mutant to induce invasive pulmonary aspergillosis, a highly significant (p<0.001) reduction in the virulence capacity of this strain was observed (Fig. 7A) with more than 80% of the cohort surviving the infection. This virulence attenuation was once again specifically ascribed to the absence of *metR*, since the reconstituted strain regained full virulence. Histological inspection of lung sections from infected animals revealed that infectious propagules of the *metRΔ* strain had been cleared in the course of infection by the residual immune system, in contrast to invasive tissue penetration of the wild-type progenitor strain. Accordingly, all lungs inspected from the *metRΔ*-infected cohort appeared as normal, while invasive growth of hyphal elements became evident for those infected with the wild-type strain. Fungal burdens assessed from lungs of infected mice (n = 5 animals per group) indicated a 50-fold reduction in colony forming units per gram tissue (500±141 *vs.* 23 849±3 770) for the deletion strain in comparison to its wild-type progenitor (Fig. 7B). To further corroborate the differences in virulence, competitive infection experiments were performed in order to obtain a competitive index (CI) [13]. In this assay, a cohort of four animals was infected with an input ratio of 1∶1 for wild-type and the deletion mutant and, four days later, output ratios were determined by assessing the number of colony forming units on permissive and selective media from homogenised pulmonary tissue. A mean CI value smaller than 0.1 was calculated for the *metRΔ* mutant (Fig. 7C), meaning that this strain is virtually avirulent. To finally analyse the dissemination capacity of the *metRΔ* mutant in the bloodstream of leukopenic mice and the relevance of sulphur utilization for this process, a systemic infection model was applied (Fig. 7D). Animals infected intravenously with the mutant strain showed significantly delayed mortality (p<0.001). In the light of these results we conclude that regulation of sulphur assimilation is essential for manifestation of pulmonary aspergillosis as well as relevant for haematogenous dissemination after angioinvasion of *A. fumigatus*.

### A *metRΔ* deletion strain displays an imbalance in iron homeostasis

**Figure 8:**
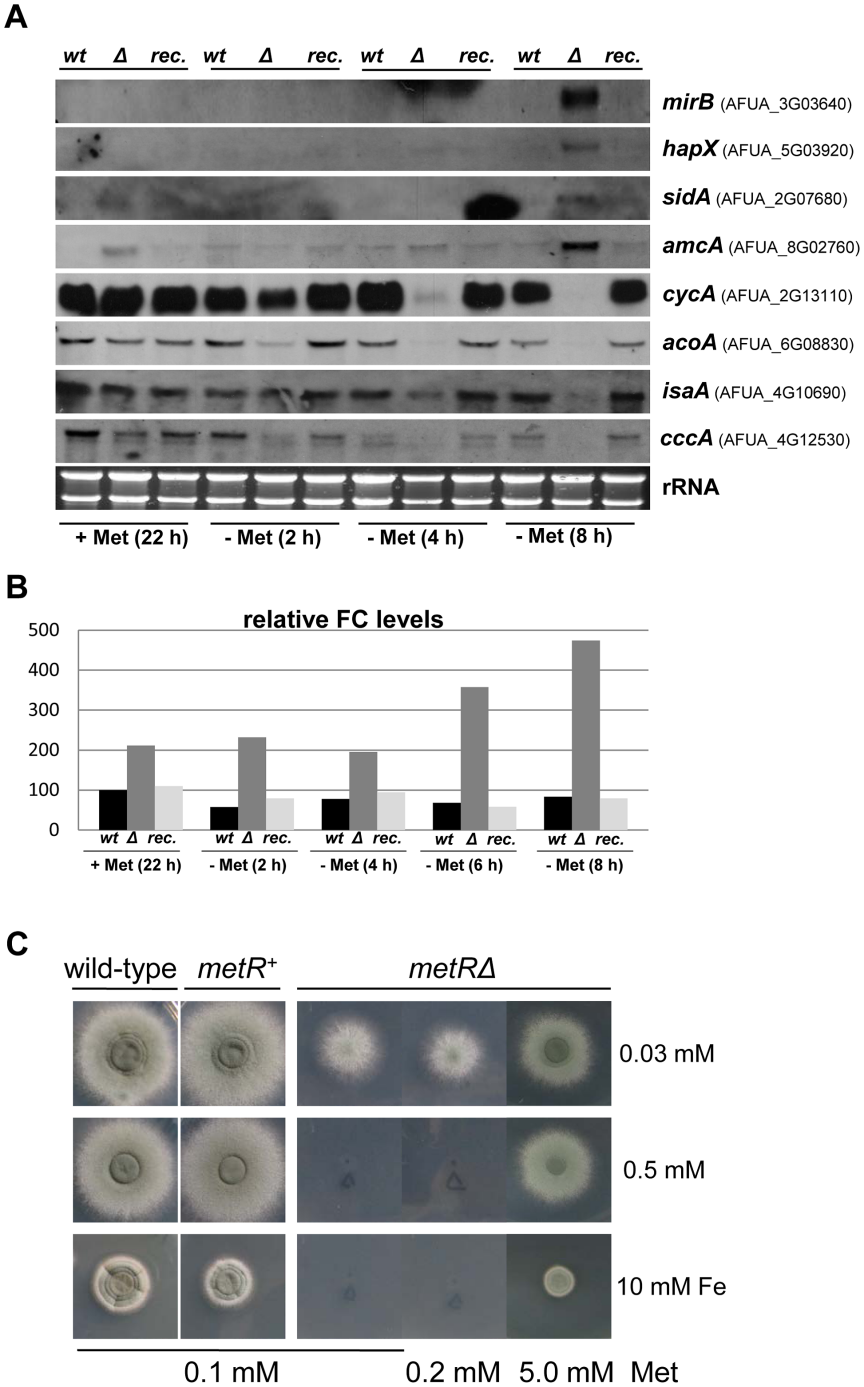
Regulatory cross-talk between sulphur and iron metabolism. (A) Fungal cultures were shifted after prolonged growth in medium containing methionine as sole source of sulphur [Met (22 h)] to medium depleted for this amino acid but containing sulphate, and samples for RNA preparation were taken after the indicated time points. Northern hybridisation expression analysis of several genes directly related with iron metabolism was carried out. Although there is sufficient iron in the medium (100 µM), after eight hours of incubation in media containing sulphate (constituting sulphur starving conditions for the mutant) increased transcription of genes typically expressed under iron-depleted conditions (*mirB*: siderophore transporter; *hapX:* transcriptional activator of the iron regulon; *sidA*: L-ornithine monooxygenase; *amcA*: mitochondrial ornithine exporter) is observed in the *metRΔ* strain as well as decreased expression of genes whose products participate in iron consuming processes (*cycA*: cytochrome C; *acoA*: aconitase; *isa1*: mitochondrial cluster biosynthetic protein). (B) Relative quantity of ferricrocin (FC) in the respective fungal mycelia. FC levels are already elevated before the shift, and they increase nearly fivefold under sulphur starvation conditions. (C) The wild-type strain is able to grow in the presence of 10 mM of iron, a substantially high concentration that already provokes toxic effects. When sufficient methionine (5 mM) is present, the *metRΔ* mutant behaves as the wild-type and the reconstituted strain. However, under sulphur starvation, the mutant is not able grow on 0.5 mM of iron or higher concentrations. This hypersensitivity might be a result of the lower expression of *cccA* gene (encoding a vacuolar iron transporter which participates in iron detoxification) in the *metRΔ* mutant, especially under sulphur starvation conditions.

It is well established that defects in mitochondrial Fe-S cluster biogenesis or transport induce transcription of the iron regulon [58], [59] and that Fe-S cluster-containing proteins participate directly in sensing iron availability in *S. cerevisiae* [60], [61]. Accordingly, we expected a regulatory cross-talk between sulphur assimilation and iron homeostasis in *A. fumigatus*, which was further indicated by the transcriptional profiling data (see above), however, which had not been tested in eukaryotes so far. We took advantage of our *metRΔ* mutant strain, which can be rapidly depleted for sulphur, to test this hypothesis. Overnight grown strains were shifted from culture media containing methionine to media depleted for this amino acid but containing sulphate. These media pose sulphur starving-conditions for the deletion strain but not for the wild-type or the reconstituted strain. After eight hours of incubation, although there was sufficient iron in the medium, the mutant strain increased transcription of several genes encoding proteins that participate in iron acquisition that are known to be upregulated under iron starvation, i.e. genes involved in siderophore biosynthesis (*sidA*), siderophore transport (*mirB*), mitochondrial ornithine export (*amcA*) and iron regulation (*hapX*) [57]. In contrast, the mutant decreased transcription of genes whose products participate in iron-consuming processes that are known to be downregulated under iron depleted conditions [40], i.e. genes encoding aconitase (*acoA*), cytochrome *c* (*cycA*) or components of the mitochondrial iron-sulfur-cluster biosynthetic machinery (*isa1*) (Fig. 8A). To analyse whether the cells were indeed depleted for iron, levels of iron chelated by ferricrocin (FC), the intracellular siderophore used for iron storage and transport [62], [63], were measured (Fig. 8B). Despite an expression pattern resembling that of iron starvation, the AfS167 mutant showed a nearly fivefold increased FC content. Combining ferricrocin analysis with total intracellular iron level measurements further underscored this imbalance in iron homeostasis (Table 4): MetR deficiency raises the cellular iron content 1.6-fold in the presence of methionine, which increases to 2.8-fold in its absence. The *metRΔ* mutant furthermore displays a 5-fold increased FC-chelated iron content under +Met conditions that is further enhanced to 7.5-fold when this S-sources is withdrawn. In conclusion, the cells indeed contain sufficient amounts of iron but display a defect in iron sensing and/or regulation. This dysregulation, causing an enhanced expression of iron uptake-related genes under sulphur starving conditions, translates into a phenotype of hypersensitivity to iron (Fig. 8C). At a low concentration of iron and methionine the *metRΔ* mutant was able to grow, although poorly due to the shortage of sulphur. This phenotype was recovered with higher availability of methionine. However, at higher iron concentrations, the wild-type strain could grow while the AfS167 mutant did not, unless a high amount of methionine was present in the medium. This might be the consequence of both the inability to shut down expression of iron uptake-related genes and of the lower *cccA* gene expression, encoding a recently described vacuolar transporter that has a prominent role in iron detoxification [64], in the *metRΔ* mutant especially under sulphur starvation (Fig. 8A), which results in iron accumulation (see Table 4: 2.4-fold increase for the *metRΔ* deletant under –Met conditions) to presumably toxic levels in the cytosol. Dysregulation of iron homeostasis was also tested in the wild-type strain by shifting its mycelium to a medium completely depleted for sulphur. However, expression of the iron regulon was not observed even after 24 hours of incubation (not shown), most likely because of the fact that the wild-type does not face such severe sulphur starving conditions as its *metRΔ* derivative apparently does.

**Table 4:** Iron contents of *A. fumigatus* in dependency of MetR and sulphur supply.

	Strains	total iron (µmol/g)*	FC^+Fe^ (µmol/g)*	non FC^+FE^ iron (µmol/g)*
	*wild-type*	1.37±0.17	0.12±0.02	1.25
+ Met	*metRΔ*	2.20±0.17 (1.6)	0.60±0.05 (5.0)	1.6 (1.3)
	*metR^+^*	1.49±0.04 (1.1)	0.38±0.04 (3.2)	1.11 (0.9)
	*wild-type*	1.84±0.18 (1.3)	0.23±0.18 (1.9)	1.61 (1.3)
− Met	*metRΔ*	3.88±0.04 (2.8)	0.90±0.10 (7.5)	2.98 (2.4)
	*metR^+^*	2.82±0.04 (2.1)	0.32±0.01 (2.7)	2.5 (2.0)

* x-fold change in comparison to *wild-type* grown with methionine is given in brackets.

## Discussion

Fulfillment of nutritional and metabolic requirements is essential for all pathogenic microorganisms to be able to grow inside the host and, thus, to cause infection and disease [7]. For opportunistic fungal pathogens this is highly relevant, since these commonly lack specific virulence factors that would provoke host damage [9]. In recent years more and more evidence has been provided that fungal metabolism is a critical component of fungal virulence [65]. Accordingly, it has been proposed that based on this knowledge novel antifungal targets might be identified [10].

Sulphur metabolism is directly related to virulence of several pathogenic microorganisms, such as *Mycobacterium tuberculosis*, *Salmonella enterica*, or protozoan parasites [66], [67], [68], [69], [70], [71]. Among fungi, sulphur metabolism has been extensively studied in the bakers' yeast *S. cerevisiae* [30], [32] and in the non-pathogenic filamentous fungi *N. crassa* [24], [28], [29] and *A. nidulans* [36]. However, our knowledge on the role of sulphur metabolism for fungal virulence has remained scarce. Only two studies have specifically addressed the importance of the sulphur-containing molecule glutathione in *C. albicans* and *C. glabrata* to demonstrate that glutathione biosynthesis, but not its uptake or degradation, is essential for virulence [23]. Accordingly, glutathione appears not to be the sulphur source these *Candida* species exploit *in vivo*, and its relevance for pathogenesis is probably due to its impact on iron metabolism [72]. For the human pathogen *Paracoccidioides brasiliensis* it was demonstrated that growth of the yeast form, which is the pathogenic state of this dimorphic fungus, strictly relies on inorganic sulphur sources and that the mycelial-to-yeast switch requires an organic source of sulphur [37], [73]. Here, we report first evidence that proper regulation of sulphur metabolism is crucial for *A. fumigatus* virulence. This result can possibly be extrapolated to other fungal pathogens and, therefore, might constitute a novel field for the identification of new targets in fighting fungal infections.

**Figure 9:**
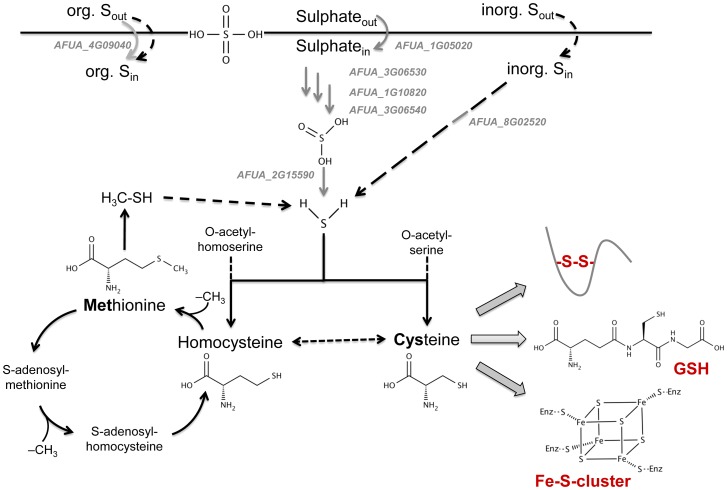
Schematic overview on the impact of MetR regulation on sulphur metabolism. The MetR regulon (gray genes and arrows) comprises genes whose products participate in sulphur assimilation, especially inorganic sulphur, rather than metabolic processes (black arrows). Full-line arrows denote known genes/pathways while dashed arrows denote putative pathways. The supposed point of connection with iron sensing/regulation is highlighted.

The information gathered in this work suggests that the MetR regulon directly affects genes whose products are related to assimilation processes of sulphur (especially inorganic sulphur), rather than metabolic processes (Fig. 9). Presence of the MetR transcription factor is essential for growth on several sulphur sources, specifically on those containing inorganic sulphur sources. Accordingly, MetR is required for activation of transcription of genes encoding enzymes of the sulphate assimilation pathway and an arylsulphatase activity, which demonstrates a direct role of MetR in inorganic sulphur acquisition. Remarkably, the *metRΔ* mutant was able to use cysteine and the Glu-Cys-Gly tripeptide glutathione as a source of sulphur only under nitrogen-starving conditions, implicating a link between S- and N-acquisition. One possible explanation is that under nitrogen-limiting conditions increased expression of amino acid permeases and oligopeptide transporters facilitates uptake of these particular sulphur-containing compounds, which then can be exploited as S-source. Since no specific enzymes for cysteine catabolism have been identified so far, this scenario could not be investigated further. The fact that the expression of the methionine aminotransferase-encoding gene *metAT* is elevated in the presence of cysteine suggests that this amino acid might be transformed into methionine rather than being catabolized directly. Accordingly, catabolism of cysteine as sulphur source appears to be MetR-independent and, therefore, its uptake might represent a bottle neck that prevents the mutant to utilize cysteine. This notion is further supported by the fact that in the RNA-seq data set expression of the oligopeptide transporter OptG (AFUA_6G03140), the orthologue to the *C. albicans* glutathione transporter *OPT7* [23], was observed to be expressed higher in the wild-type than in the *metRΔ* mutant. Thus, *cynA* and *optG* are candidate genes to support *A. fumigatus* growth in the presence of cysteine and glutathione, but further studies are needed to elucidate whether they encode specific *A. fumigatus* transporters and whether cysteine acid is catabolized directly.

The ability of *A. fumigatus* to produce volatile sulphur compounds derived from methionine catabolism has been demonstrated previously [51]. Here we demonstrate for the first time that *A. fumigatus* is furthermore able to utilize such VSCs as S-source. Importantly, we could show that utilization but not production of VSCs is MetR-dependent. This agrees with the inability of the *metRΔ* mutant to grow on S^2−^ and also with the fact that all studied genes that participate in methionine metabolism are MetR-independent in their expression. Nevertheless, further studies are necessary to unravel the methionine catabolism pathway in order to understand the capacity of a *metRΔ* mutant to utilize it as S-source, the VSCs production process, and also to identify any specific VSCs that can be utilized.

As an important part of our current study we could show that MetR is important for virulence of *A. fumigatus* in *G. mellonella* larvae as well as in leukopenic mice. In the wax moth larvae, *metRΔ* regained virulence when supplemented with methionine, suggesting that the main reason for the decrease in virulence is the absence of a suitable source of sulphur in the larval hemocoel. In the same way one might speculate that the decrease in virulence observed in the mouse model is also due to insufficient levels of methionine in the murine lung or blood and, thus, that this amino acid is not the primary source of sulphur exploited by *A. fumigatus* within the pulmonary tissue or in the bloodstream. In line with this are data from Purnell (1973) on a methionine-requiring mutant of *A. nidulans* that displayed unaltered virulence in systemic infections of mice [74]. Inorganic compounds that cannot be assimilated by the *metRΔ* mutant may accordingly serve as initial S-source during infection. Taking into account that the mammalian lung probably constitutes a nitrogen-limiting environment [17], cysteine and glutathione also likely do not serve as sources of sulphur during pulmonary infection, since the mutant is able to utilize these compounds *in vitro* under nitrogen-starving conditions. However, since several other sulphur-related processes are deregulated in the *metRΔ* mutant, we cannot conclude that the mere absence of a suitable sulphur source impairs the growth of the *metRΔ* mutant within the murine lung. Defects in iron regulation or sensing that are characteristic for the *metRΔ* mutant may also account for its attenuated virulence, since iron relates to fundamental cellular processes such as respiration or oxidative stress resistance [40], [75].

In the bakers' yeast the Aft1p and Aft2p transcription factors mediate upregulation of the so-called iron regulon under iron limiting conditions [76], [77], [78]. Iron sensing by Aft1p and Aft2p requires proper mitochondrial Fe-S cluster biosynthesis as well as a functional export to the cytoplasm and, consequently, disturbance of these processes provokes upregulation of the iron regulon [58], [59], [79]. Impairment of Fe-S cluster biogenesis can be achieved by disruption of the cysteine desulphurase-encoding gene *nfs1* that is required for sulphide supply in Fe-S cluster biosynthesis [80], [81]. In addition, it was shown that glutathione participates in Fe-S cluster translocation to the cytoplasm and thus its depletion activates the iron regulon [79], [82]. All these relations between sulphur-containing molecules and iron-dependent transcriptional regulation strongly suggest a connection between sulphur metabolism and iron homeostasis. Other fungi, such as *Aspergillus* species or *Schizosaccharomyces pombe*, do not express Aft1/2 orthologues. Here, iron regulation is mediated by the interplay of the unrelated HapX/Php4 and SreA/Fep1 proteins [40], [83], [84]. Yet, the strategy for iron sensing is likely to be conserved in the fungal kingdom, which might link iron homeostasis to sulphur metabolism in general. Here, by virtue of a deletion mutant, we clearly demonstrate this relationship: starving the *A. fumigatus metRΔ* mutant strain for sulphur results in increased expression of the iron regulon. We hypothesize that the pronounced sulphur starvation of the *metRΔ* mutant impairs Fe-S cluster biogenesis and/or glutathione biosynthesis, which, in turn, activates the iron regulon. The corresponding wild-type isolate would not act on iron homeostasis under sulphur-depleting conditions as it apparently does not face such a severe starvation, most likely due to the utilization of reserve pools and salvage pathways. We cannot rule out the possibility that MetR directly regulates transcription of an unidentified gene whose product is required for iron sensing or proper iron regulation under sulphur-starving conditions. But given the fact that MetR deficiency strikingly phenocopies a deficiency for the negative iron regulator SreA (increased cellular iron as well as ferricrocin contents accompanied by transcriptional derepression of genes involved in iron acquisition such as siderophore biosynthesis and uptake as well as reductive iron assimilation), such a direct action on the *sreA* and *hapX* genes encoding the main players of iron homeostasis in *A. fumigatus* is unlikely. Along that line, we could not detect binding of the MetR-GFP protein to promoter regions of either gene by chromatin immune-precipitation. The disclosed link between sulphur metabolism and iron homeostasis represents an appealing crosstalk between two fundamental cellular regulatory circuits that calls for further investigation.

In summary, we show for the first time that regulation of sulphur metabolism is important for the ability of *A. fumigatus* to cause disease. Given the conserved nature of sulphur assimilation in the fungal kingdom, its relevance in virulence is likely to be a general feature among pathogenic fungi. Considering that many of these routes are absent in mammals, some of these processes might represent suitable novel targets for antifungal drug development.

## Materials and Methods

### Ethics statement

Mice were cared for in accordance with the principles outlined by the European Convention for the Protection of Vertebrate Animals Used for Experimental and Other Scientific Purposes (European Treaty Series, no. 123; http://conventions.coe.int/ Treaty/en/Treaties/Html/123.htm). All infection experiments were carried out in compliance with the German animal protection law in a protocol approved by the Government of Lower Franconia (file number: 55.2-2531.01-90/09).

### Strains, media and culture conditions

The *Escherichia coli* strain DH5α [85] was used for cloning procedures. Plasmid-carrying *E. coli* strains were routinely grown at 37°C in LB liquid medium (1% peptone, 0.5% yeast extract, 0.5% NaCl) under selective conditions (100 µg•ml^−1^ ampicillin or 50 µg•ml^−1^ kanamycin); for growth on plates, 1.5% agar was added to solidify the medium. All plasmid constructs used in the course of this study are listed in Table S2 and were generated using the Seamless Cloning (Invitrogen) technology as described in the Supplementary Material (Text S1).

The wild-type *A. fumigatus* strain ATCC 46645 served as common reference [86], derivatives of this isolate generated in the course of this study are described in the Supplementary Material (Text S2). *A. fumigatus* strains were basically cultured in nitrate-based minimal medium [87] containing 1% glucose, 70 mM NaNO_3_, 7 mM KCl, 11 mM KH_2_PO_4_ (pH 5.5), 0.25 mM MgSO_4_, trace elements solution, and 2% agar (Serva) for solid media at 37°C. In case of selection for the presence of the hygromycin B resistance marker, 50 µg•ml^−1^ of this antibiotic (InvivoGen) were applied. In sulphur-free medium, MgCl_2_ was substituted for MgSO_4_ and a modified mixture of trace elements lacking any sulphate salt was used. For preparation of porcine lung agar (PLA) culture medium, 5 g of fresh tissue was snap-frozen in liquid nitrogen and pulverized using a pre-cooled mortar. The resulting powder was put into a 50 ml reaction tube, filled up with an equal amount of sterile saline and briefly incubated in a 50°C warm water bath, followed by addition of an equal amount of 50°C warm, liquid water agar (1.5% agarose in water). To suppress bacterial growth, PLA media was supplemented with 50 µg•ml^−1^ tetracycline. The suspension was finally vortexed for 10 s and poured onto solidified water agar.

For all growth assays on solid media, the culture medium was inoculated with 10 µl of a freshly prepared *A. fumigatus* spore suspension (10^5^ conidia•ml^−1^ in water supplemented with 0.9% NaCl and 0.02% Tween 80) and incubated at 37°C for three days. Phenotypic microarray plates (Biolog PM 4) were inoculated as follows: a suspension containing 1.5×10^6^ spores•ml^−1^ in sulphur-free minimal medium was prepared and 100 µl aliquots of this were added to each well. Fungal growth was measured at 48 hours by optical density (O.D.) at 630 nm in a Multiskan Ascent microplate photometer (Thermo Electron).

For the measurement of germination percentages, 10^7^ conidia were inoculated in 200 ml sulphur-free minimal media supplemented with 2 mM sulphate or 5 mM methionine and incubated at 37°C and 150 r.p.m for 11 hours. Each hour a 1 ml aliquot was taken, sonicated, and the germination percentage calculated as the ratio of germinated conidia with respect to the total number of spores.

*A. fumigatus* liquid media shifts were performed according to Narendja *et al.* [88] with adjusted media to modify the sources of sulphur: 200 ml minimal medium lacking sulphur and supplemented with 5 mM methionine were inoculated with 10^8^ freshly harvested, 5-day-old *A. fumigatus* ATCC 46645, *metRΔ*, or *metR^+^* conidia and propagated at 37°C and 150 r.p.m. for 16 to 22 hours. Mycelia from such pre-cultures were then harvested, washed extensively with water, and split into similar aliquots on a sterile surface. These were then added to 100 ml of minimal medium base without sulphur source or supplemented with 2 mM SO_4_^2−^, 5 mM methionine, or 5 mM cysteine, respectively, and incubated at 37°C and 150 r.p.m for one to eight hours.

### Extraction and manipulation of nucleic acids

Standard protocols of recombinant DNA technology were carried out [89]. Phusion high-fidelity DNA polymerase (Fermentas) was generally used in polymerase chain reactions and essential cloning steps were verified by sequencing. Fungal genomic DNA was prepared following the protocol of Kolar *et al.* [90] and Southern analyses were carried out as described [91], [92]. Probes for non-radioactive hybridizations were generated and detected using the Gene Images AlkPhos Direct Labelling and Detection System from GE Healthcare. Samples of total RNA were isolated with the TRIzol reagent (Sigma) and cleaned with peqGOLD phase-trapA (peqlab). RNA samples for RNA-seq were further purified with RNeasy Plant Mini Kit columns (Qiagen). For Northern hybridisation analyses, 10 µg of total RNA were separated in formaldehyde-containing agarose gels, blotted onto Hybond-N^+^ membranes (Amersham Biosciences), and hybridized with digoxigenin-labeled probes prepared as recommended by the manufacturer (Boehringer Mannheim). Templates for hybridization probes were generated by PCR amplification using oligonucleotides listed in Table S3. Autoradiographies were produced by exposing washed membranes to Fujifilm RX films.

### Chromatin immunoprecipitation

Binding of MetR to promoter regions of selected candidate targets was interrogated following the chromatin immunoprecipitation (ChIP) approach together with the GFP-Trap technology (ChromoTek) and using strain AfS171 that carries a codon-optimised version of the *gfp2-5* allele [93] from pSK494 [94] preceded by a (GA)_5_ linker region [95] fused C-terminally to the *metR* coding sequence. Essential steps were carried out following the protocol of [96] with modifications (see *Supporting Information* for details), enrichment for distinct fragments was probed by semi-quantitative PCRs with specific primer pairs covering respective promoter regions.

### Transcriptional profiling

Digital transcriptomes of *A. fumigatus* strains ATCC 46645 and AfS167 [*metRΔ*] were produced by Eurofins MWG Operon GmbH from two independent biological replicates, which underlay the high reproducibility of this experimental approach [97]. For this purpose, 3′-fragment-specific cDNA libraries were prepared from poly(A)-fragment selected mRNA and processed on the Illumina HiSeq 2000 sequencing system using v3.0 chemistry and the 1×100 bp single read module, which ensures a high significance in respect to the copy number of each transcript. Mapping of reads on the most recent reference genome sequence (http://www.aspergillusgenome.org/) was performed using BMA, SamTools and Picard software. To enable the direct comparison between the samples, the read count per reference has been normalized as follows: (total_mapped_reads_per_refseq/number_of_reads_in_sample)*lowest_total_sample_read_count and differential expression analyses were carried out using DESeq [98]. A 1.5-fold change between the average number of reads in the wild-type and *metRΔ* strain was used as threshold to define genes which are expressed higher or lower, respectively, in the mutant. Functional characterization of these regulated genes was performed on the FungiFun website (https://sbi.hki-jena.de/FungiFun/FungiFun.cgi) [55] based on both the FunCat method and the Gene Ontology (GO) classification [99].

### Virulence models for aspergillosis

Infections of larvae from the greater waxmoth *Galleria mellonella* were performed according to Kavanagh & Fallon [100]. Larvae were injected with varying doses of conidia in a saline solution supplemented with 0.02% Tween 80 and 10 µl•ml^−1^ rifampicin, to avoid bacterial infections, and incubated at 30°C.

Female mice (CD1 or BALB/c from Charles Rivers Breeding Laboratories, Sulzfeld, Germany) of 20 to 24 g were used for infection experiments. Immunosuppression was carried out by subcutaneous injection of 112 mg•kg^−1^ hydrocortisone acetate and intraperitoneal injection of 150 mg•kg^−1^ cyclophosphamide following a sequential protocol as previously described [101], with the modification that two doses of cortisone on days −3 and −1 were applied. Bacterial infections were prevented by adding 2 g•l^−1^ neomycin to the drinking water. Inocula were prepared by harvesting conidia from 5-day-old slants of solid medium followed by filtration through Miracloth tissue and washing with saline. Mice were anesthetized by intraperitoneal injection of a ketamine (1%)/xylazine (0,2%) solution and either infected intranasally by instillation of 2•10^5^ conidiospores suspended in 40 µl of saline or intravenously by injection of a 50 µl suspension of 1•10^5^ conidia into the lateral tail vein. Disease progression was followed twice daily by tabulating weight profiles and following the animals' behaviour. Signs of respiratory distress, hunched posture or poor mobility, as well as severe weight loss of more than 20% determined the experimental end point for each animal. To evaluate mortality rates in single-strain infection experiments, the log rank method was applied using the GraphPad Prism software.

For competitive index (CI) assessment [13], mice were intranasally infected with a 1∶1 mixture of 2•10^4^ conidia from ATCC 46645 and the *metRΔ* strain AfS167 and sacrificed after four days. The lungs were explanted and aliquots from homogenized tissue were spread onto media containing or lacking methionine as sulphur source to differentiate between the wild-type progenitor and its *metRΔ* deletant. The CI is defined as the output ratio of mutant to wild-type fungal colonies divided by the input ratio of mutant to wild-type fungal colonies [102].

Histological cryo-sectioning was performed on 4% formaldehyde-fixed lungs, staining procedures with hemotoxylin and eosin together with Grocott's Methenamine Silver were carried out according to standard protocols. Five sections each from four lungs of mice infected with the wild-type and *metRΔ* strain, respectively, were inspected to yield representative images.

### Ferricrocin and iron content measurements

Quantification of the intracellular siderophore ferricrocin (FC) was carried out as described earlier [40], [103]. Samples for total iron content measurements were lyophilized and 50 mg digested in 500 µl 60% HNO_3_ (Ultrapure, Merck) for 4 h at 110°C and diluted thereafter with ultrapure water (Milli-Q). Iron was quantified by graphite furnace atomic absorption spectrometry (M6 Zeeman GFAA-Spectrometer, Thermo Scientific) at 248.3 nm and D2-Quadline background correction using 1000°C ash temperature and 2100°C atomization temperature under argon atmosphere. The iron content was calculated by interpolation from an appropriate standard curve (0.5 to 12.5 µg•l^−1^) using TraceCERT (Sigma-Aldrich) standard solution. The accuracy of analysis was assessed by simultaneous analysis of a standard reference human serum sample (ClinChek, Recipe).

## Supporting Information

Figure S1 **Deletion and reconstitution of the** ***metR*** **gene.** (A) The complete *metR* ORF was replaced *via* homology recombination by a blaster cassette containing the hygromycin B resistance gene as selectable marker. Correct integration was checked by Southern blot hybridisation. Afterwards, the cassette was removed as a result of the action of the β-recombinase included in the cassette itself, expression of which is driven by a xylose-inducible promoter. Correct excision of the cassette was also checked by Southern analysis. (B) The *metR* gene was reintroduced at its original locus using its own 5′and 3′flanking sequences as homology regions. A silent punctual mutation was inserted to create an extra *BstE*II restriction site to allow differentiation between the reconstituted strain and its progenitor. Selection was performed by recovery of the sulphate utilization capacity, and correct integration was checked by Southern hybridisation. In both strategies the used probe is marked and only relevant restriction sites are shown.
(TIF)

Figure S2 **Phenotypical characterisation of the** ***metRΔ*** **deletant with respect to various sources of nitrogen, carbon, or phosphorus.** Conidia of the *metRΔ* deletion strain, its wild-type progenitor, and the reconstituted derivative were point inoculated on culture plates containing either methionine or sulphate as S-source and that were supplemented with the indicated sources of nitrogen, carbon, or phosphorus. Only for the carbon source galactose a pronounced influence on methionine utilisation became evident, whereas all other N-, C-, or P-sources have no influence on growth capacities of the deletant.
(TIF)

Figure S3 **The** ***metRΔ*** **strain displays reduced virulence in an alternative infection model.** Larvae of the greater wax moth *Galleria mellonella* (n = 15 insects per group) were infected with conidial suspensions and survival was monitored. The *metRΔ* mutant shows a significant reduction in virulence, comparable to the established, avirulent *pabaAΔ* control strain. When injected in a solution containing 5 mM methionine, the mutant regained its ability to kill the larvae. The reconstituted strain recovered full virulence. Control mice either received no injection (‘untreated’), were pricked, but not injected (‘puncture’) or were mock injected using the solvent alone (‘NaCl/Tween’).
(TIF)

Table S1 **RNAseq data.**
(XLS)

Table S2 **Plasmids used in the course of this study.**
(DOC)

Table S3 **Oligonucleotides used in this study.**
(DOC)

Text S1 **Construction of plasmids and recombinant** ***A. fumigatus*** **strains and chromatin immunoprecipitation (ChIP) protocol for identification of MetR targets.**
(DOC)
