# Supplementary figures and images for: Regulation of Sulphur Assimilation Is Essential for Virulence and Affects Iron Homeostasis of the Human-Pathogenic Mould Aspergillus fumigatus
Source: PLoS Pathog. 2013 Aug 29;9(8):e1003573. doi: 10.1371/journal.ppat.1003573 (PMC3757043; doi:10.1371/journal.ppat.1003573)

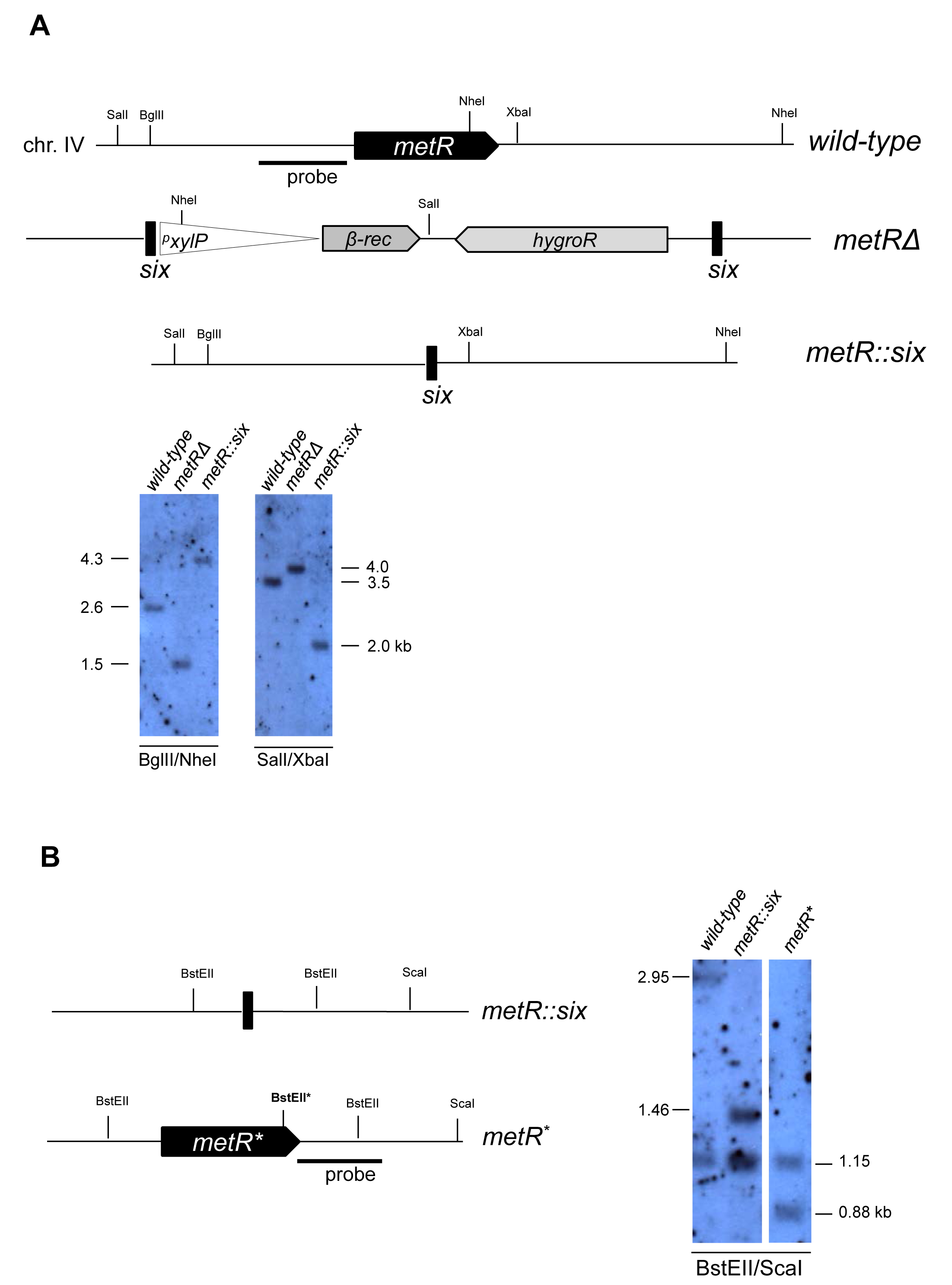

Supplement: Figure S1 — Deletion and reconstitution of the metR gene. (A) The complete metR ORF was replaced via homology recombination by a blaster cassette containing the hygromycin B resistance gene as selectable marker. Correct integration was checked by Southern blot hybridisation. Afterwards, the cassette was removed as a result of the action of the β-recombinase included in the cassette itself, expression of which is driven by a xylose-inducible promoter. Correct excision of the cassette was also checked by Southern analysis. (B) The metR gene was reintroduced at its original locus using its own 5′and 3′flanking sequences as homology regions. A silent punctual mutation was inserted to create an extra BstEII restriction site to allow differentiation between the reconstituted strain and its progenitor. Selection was performed by recovery of the sulphate utilization capacity, and correct integration was checked by Southern hybridisation. In both strategies the used probe is marked and only relevant restriction sites are shown. (TIF) [file ppat.1003573.s001.tif]

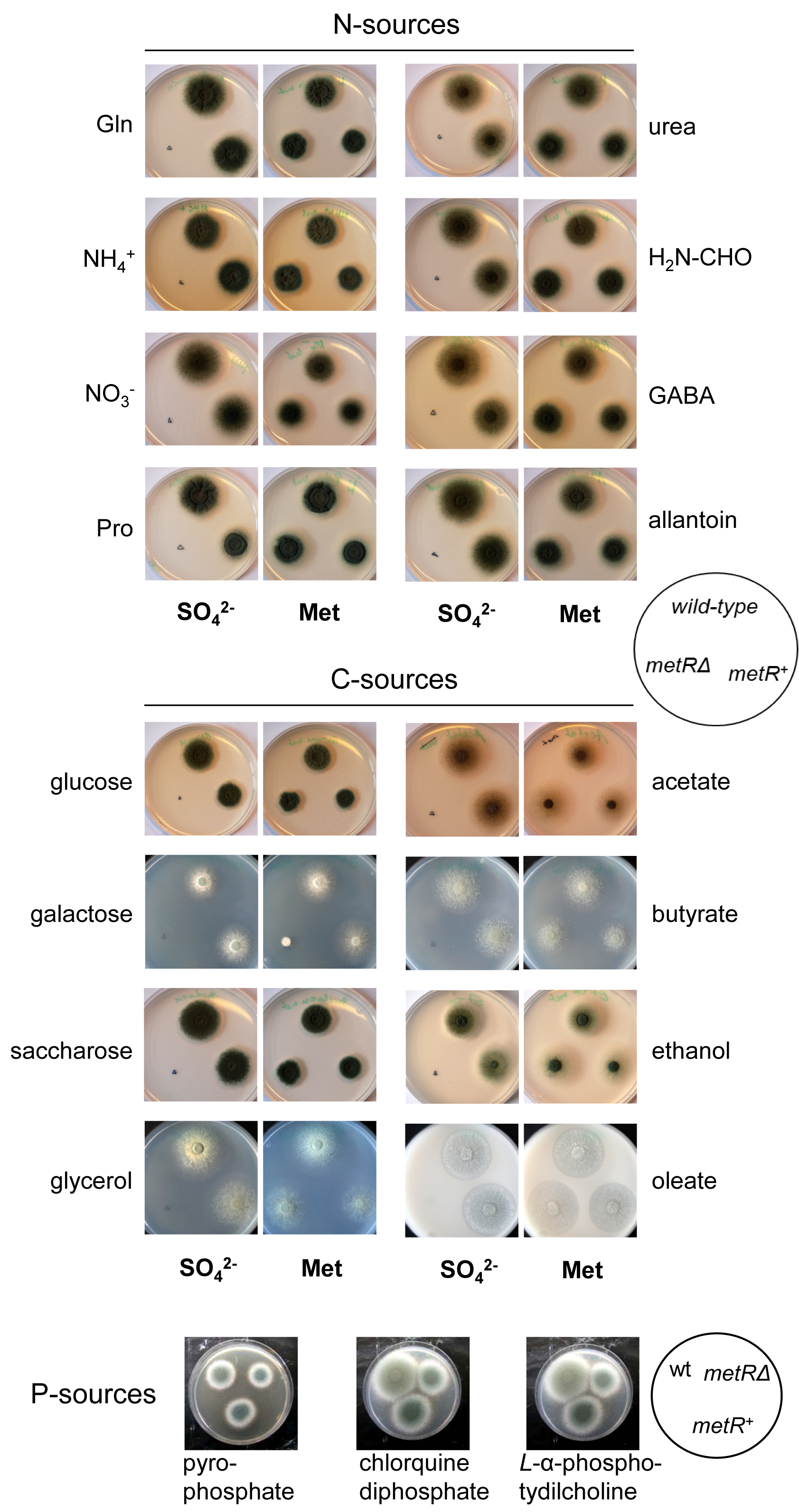

Supplement: Figure S2 — Phenotypical characterisation of the metRΔ deletant with respect to various sources of nitrogen, carbon, or phosphorus. Conidia of the metRΔ deletion strain, its wild-type progenitor, and the reconstituted derivative were point inoculated on culture plates containing either methionine or sulphate as S-source and that were supplemented with the indicated sources of nitrogen, carbon, or phosphorus. Only for the carbon source galactose a pronounced influence on methionine utilisation became evident, whereas all other N-, C-, or P-sources have no influence on growth capacities of the deletant. (TIF) [file ppat.1003573.s002.tif]

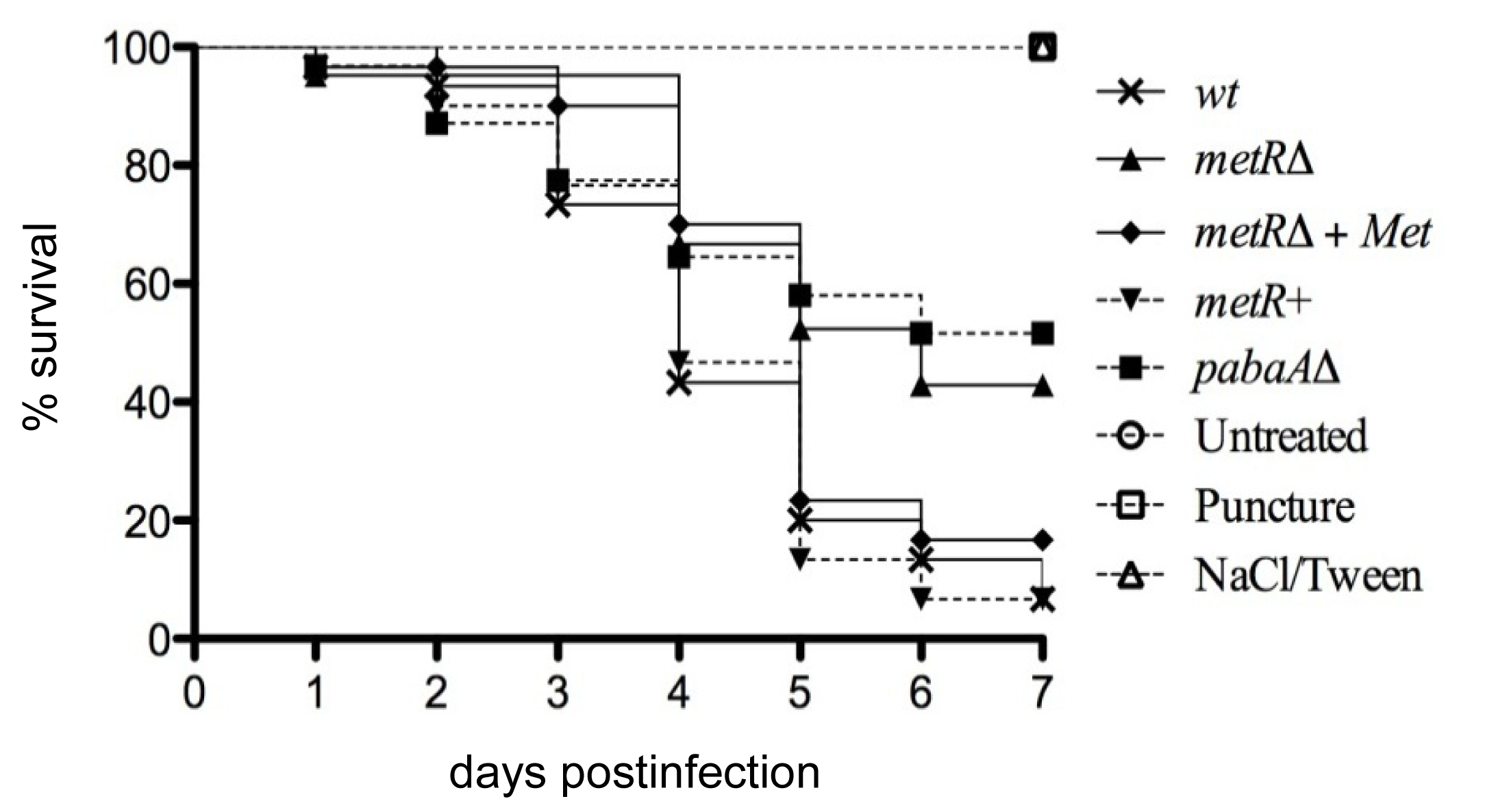

Supplement: Figure S3 — The metRΔ strain displays reduced virulence in an alternative infection model. Larvae of the greater wax moth Galleria mellonella (n = 15 insects per group) were infected with conidial suspensions and survival was monitored. The metRΔ mutant shows a significant reduction in virulence, comparable to the established, avirulent pabaAΔ control strain. When injected in a solution containing 5 mM methionine, the mutant regained its ability to kill the larvae. The reconstituted strain recovered full virulence. Control mice either received no injection (‘untreated’), were pricked, but not injected (‘puncture’) or were mock injected using the solvent alone (‘NaCl/Tween’). (TIF) [file ppat.1003573.s003.tif]
