# Supplementary material for: Regulation of Sulphur Assimilation Is Essential for Virulence and Affects Iron Homeostasis of the Human-Pathogenic Mould Aspergillus fumigatus
Source: PLoS Pathog. 2013 Aug 29;9(8):e1003573. doi: 10.1371/journal.ppat.1003573 (PMC3757043; doi:10.1371/journal.ppat.1003573)
Supplement: Table S2 — Plasmids used in the course of this study. (DOC) [file ppat.1003573.s005.doc]

**Table S2**

Plasmids used in the course of this study

| Plasmid | Description | Reference |
| --- | --- | --- |
| pUC19L | general cloning vector | Invitrogen |
| pSK494 | codon-optimised version of *gfp2-5* allele | [94] |
| pSK529 | β-rec/*six* blaster module including hygromycin B resistance cassette | [46,47] |
| pSK574 | *A. fum. metR∆* replacement cassette: β‑rec/*six* blaster module from pSK529 flanked by 5´and 3´*metR* homology regions | this study |
| pSK575 | *A.fum. metR* reconstitution cassette: *metR^+^* locus with a silent C→G punctual mutation at pos. 1328 | this study |
| pSK583 | replacement cassette carrying *metR::GA_5_::gfp2-5* allele | this study |
