# Supplementary material for: Regulation of Sulphur Assimilation Is Essential for Virulence and Affects Iron Homeostasis of the Human-Pathogenic Mould Aspergillus fumigatus
Source: PLoS Pathog. 2013 Aug 29;9(8):e1003573. doi: 10.1371/journal.ppat.1003573 (PMC3757043; doi:10.1371/journal.ppat.1003573)
Supplement: Table S3 — Oligonucleotides used in this study. (DOC) [file ppat.1003573.s006.doc]

**Table S3**

***Oligonucleotides used in this study***

| Oligonucleotide | Sequence 5´→3´ | Purpose |
| --- | --- | --- |
| Sv746 | AATTCGAGCTCGGTACTGCGCACAAGGAATCGGATTACGAG | pJA1 seamless cloning / *metRΔ* Southern probe (forward) |
| Sv747 | GGACCTGAGTGATGCCTTCTTCAAATGCGTCC | pJA1 seamless cloning / *metRΔ* Southern probe (reverse) |
| Sv748 | TGGTCCATCTAGTGCGTGAGCAGAGCCAAATGAAAAAG | pJA1 seamless cloning / *metR* reconstitution Southern probe(fw) |
| Sv749 | GCCAAGCTTGCATGCCTGCGCAGCGACGATAACGGTGTGT | pJA1 seamless cloning / *metR* reconst. Southern probe (rev) |
| Sv837 | AATTCGAGCTCGGTACGTTAACCCCTAGTTTTCGTTGAAAAAGC | pJA20 cloning (fw) |
| Sv838 | CGGTCACCCGGGCCTCAAGAGCG | pJA20 cloning (rev) |
| Sv839 | AGGCCCGGGTGACCGCGCTGGAGC | pJA20 cloning (fw) |
| Sv840 | GCCAAGCTTGCATGCCGTTAACCGCCTTACTTCGTGACAGATGG | pJA20 cloning (rev) |
| Sv863 | AATTCGAGCTCGGTACGTTAACATGCTCTCTGATGAAGGTCTCG | *metR::GA_5_::gfp2-5* cloning |
| Sv864 | GCCTGCACCAGCTCCCGCAGTGCCAACACCGATCTTGG | *metR::GA_5_::gfp2-5* cloning |
| Sv865 | GGAGCTGGTGCAGGCGCTGGAGCCTCCAAGGGCGAGGAACTGTTCACC | *metR::GA_5_::gfp2-5* cloning |
| Sv866 | CTTGTACAGCTCGTCCATGCCGTGG | *metR::GA_5_::gfp2-5* cloning |
| Sv867 | GACGAGCTGTACAAGTGAGCAGAGCCAAATGAAAAAGAAG | *metR::GA_5_::gfp2-5* cloning |
| Sv868 | GCCAAGCTTGCATGCCGTTAACCTGGTTGTTCTGGTTGTGTCC | *metR::GA_5_::gfp2-5* cloning |
| Sv877 | CTGTCAGCAGTAGCAGTACC | *sC* ChIP semi-qPCR |
| Sv878 | AGATAGCGAGTCGGTGATCG | *sC* ChIP semi-qPCR |
| Sv881 | CACGCAAGCTCTGATGATCG | *sD* ChIP semi-qPCR |
| Sv882 | GGAGGAAGAAGAGATGATGCC | *sD* ChIP semi-qPCR |
| Sv903 | TCCACCCAATAGAGCTGGCG | arylsulph. ChIP semi-qPCR |
| Sv904 | CTGGCGATCTCACATCTCACC | arylsulph. ChIP semi-qPCR |
| Sv919 | CATTAGCTCCAGTTCCCTTGC | *hapX* ChIP semi-qPCR |
| Sv920 | GATTACGGATGATGAGACTCCC | *hapX* ChIP semi-qPCR |
| JAE106 | AACTTCTCTCAATACCTCGAGG | *metR* Northern probe (Np) (fw) |
| JAE107 | TCTTGGACTCGGAACTACTCCG | *metR* Np (rev) |
| JAE108 | GTTTCTCGGGGATGGAGTGC | *sB* ChIP semi-qPCR |
| JAE109 | GGTAGATAGAGGAAGGGATGG | *sB ChIP* semi-qPCR |
| JAE118 | TGACCATCCACTCGGTCATCG | *sB* Np (fw) |
| JAE119 | GCATTCACCTCAGCAGGATCG | *sB* Np (rev) |
| JAE187 | CACCAAGCCTGGAGACATCG | *sC* Np (fw) |
| JAE188 | GATGATCTCGTGGACGATGC | *sC* Np (rev) |
| JAE185 | ACATCACCTACCACGCCAGC | *sD* Np (fw) |
| JAE186 | CCCCTGCTCATCCAGATACG | *sD* Np (rev) |
| JAE128 | CAGGATGCCTGCCAAGTTGC | *sA* Np (fw) |
| JAE129 | TCTACGCAGTGGTGAGCTGG | *sA* Np (rev) |
| JAE126 | ACCTGCTTCCTGCCACTACC | sulphite Reductase Np (fw) |
| JAE127 | ACCAGTCTCCGAATCGCTCC | sulphite Reductase Np (rev) |
| JAE130 | AACTCATGAACGGCCACACG | arylsulphatase Np (fw) |
| JAE131 | GAACCATCAGTAGTGCGTCC | arylsulphatase Np (rev) |
| JAE71 | TTGATGCGACACCAGACGACC | *mupA* Np (fw) |
| JAE72 | GGACGAAGTATGTTCAAGCTCG | *mupA* Np (rev) |
| JAE75 | TACGAGTCGATCCACGACATCC | *mupC* Np (fw) |
| JAE76 | ACCCAGGCAAACGTGACTACC | *mupC* Np (rev) |
| JAE77 | CTCCTCAGATTCGTGACTGAGC | Met Aminotransferase Np (fw) |
| JAE78 | GGCAGGATGCTTCTGCCAATCG | Met Aminotransferase Np (rev) |
| JAE65 | CTCTCGTGATGAGCTCTTGAGC | *metH* Np (fw) |
| JAE66 | GACAGCGAAGCTGAACCACTCC | *metH* Np (rev) |
| JAE134 | CATCGACCGCTTACCATGAGC | *cysB* Np (fw) |
| JAE135 | CTTCCACTTCGCTCGATCAGG | *cysB* Np (rev) |
| JAE69 | AGTCGTCCGCAATGTCTTCTGG | *cynA* Np (fw) |
| JAE70 | CTATAGGAACAGCCACATCCC | *cynA* Np (rev) |
| oAfmirB1me | AAGCCGAGAAAAAGGGGG | *mirB* Np (fw) |
| oAfmirB1me | AACCCAGATGAAGCCCAG | *mirB* Np (rev) |
| oHapX-seq1 | TCGGTGGAAAGAAGTGCC | *hapX* Np (fw) |
| oHapX-seq2 | CGAGTCCGTTTGGGTATC | *hapX* Np (rev) |
| osidA1 | ACTACCTCCACCAGAAGG | *sidA* Np (fw) |
| osidA2 | ACCTTGAAGCCAGATGCC | *sidA* Np (rev) |
| oAfAmcA-f | TCAATGGAGCTGCCTGTC | *amcA* Np (fw) |
| oAfAmcA-r | CAATTCCGTAGCCCTTCG | *amcA* Np (rev) |
| oAfcytC1 | CCCTTTCTTGCAGTGTCC | *cycA* Np (fw) |
| oAfcytC2 | CCGCGCATCTGCTTTTAC | *cycA* Np (rev) |
| oAfacoA1 | CAGCGTCCTCTCACATAC | *acoA* Np (fw) |
| oAfacoA2 | GCAAGAACCGATCAGACC | *acoA* Np (rev) |
| oAfIsa1.f | CAGCTACTGCATACCGAC | *isa1* Np (fw) |
| oAfIsa1.r | AGACCATGAAGGATTCGC | *isa1* Np (rev) |
