# Supplementary material for: Regulation of Sulphur Assimilation Is Essential for Virulence and Affects Iron Homeostasis of the Human-Pathogenic Mould Aspergillus fumigatus
Source: PLoS Pathog. 2013 Aug 29;9(8):e1003573. doi: 10.1371/journal.ppat.1003573 (PMC3757043; doi:10.1371/journal.ppat.1003573)
Supplement: Text S1 — Construction of plasmids and recombinant A. fumigatus strains and chromatin immunoprecipitation (ChIP) protocol for identification of MetR targets. (DOC) [file ppat.1003573.s007.doc]

**Text S1.**

*Construction of plasmids and recombinant A. fumigatus strains.*

For construction of plasmids the fragments of interest were obtained as described below and assembled in one step in the pUC19L vector using the Seamless Cloning technology (Invitrogen). For this protocol, overlapping 15 bp homology regions were designed at the extremes of each fragment to specifically assemble them in the vector in the correct order by virtue of a recombination process.

For construction of pSK574, the replacement cassette, containing the hygromycin B resistance gene and the β-recombinase-encoding gene under the control of the xylose-inducible promoter, was obtained by digestion of the pSK529 plasmid with the *Fsp*I restriction enzyme. Homology regions 5´and 3´of *metR* were amplified by PCR using primers Sv746/747 and Sv748/749 respectively. These three fragments were assembled in the pUC19L vector as explained above and correct recombination was verified by multiple restriction analysis. For construction of pSK575, two rounds of PCR amplification were performed. The first one, amplifying 1521 bp upstream of *metR* gene plus nucleotides 1→1335 from *metR* ORF, was performed with primers Sv837/838; the second one, comprising nucleotides 1320-1517 of the *metR* coding sequence plus 1729 bp downstream, with primers Sv839/840. In the Sv838-Sv839 overlapping region a silent 1328^C→G^ mutation was introduced to create an additional *BstE*II restriction site. These amplicons were assembled in the pUC19L vector and correct recombination was verified by multiple restriction analysis. Once the plasmid had been constructed, the regions amplified by PCR were sequenced to avoid undesirable mutations. Transformation of the *A. fumigatus* wild-type ATCC 46645 with plasmid pSK574 resulted in the *metRΔ* deletion strain AfS166 [*metR::six-^p^xylP::β-rec::trpC^t^; hygro^R^-six*] which expresses a hygromycin B resistance cassette and the β‑recombinase gene driven by a xylose-inducible promoter. Subsequent growth of this strain on xylose-containing medium resulted in excision to result in the *metR::six* strain AfS167, which is also designated as *metRΔ*. Transformation of the *metR::six* strain with plasmid pSK575 yielded strain AfS168 [*metR^C1328→G^*], indicated as *metR^+^* in subsequent experiments.

The replacement cassette for reconstitution of the *metRΔ* deletion strain with a functional *metR::gfp* allele as assembled *via* Seamless Cloning of three fragments covering the *metR* 5' region together with the coding sequence amplified from *A. fumigatus* genomic DNA with oligonucleotides Sv863/864, a codon-optimised version of the *gfp2-5* allele amplified with Sv865/866 from pSK494 and preceded by a (GA)_5_ linker region, and the *metR* 3' untranslated region (Sv867/868). The resulting 5.4 kb fragment of pSK583 was released from the pUC19 backbone by *Hpa*I digest and transformed in the *metRΔ* recipient. Transformants were selected by their capacity to utilize sulphate as source of sulphur and verified for integration of the *metR::gfp* allele at the original locus by diagnostic PCR on genomic DNA. One representative isolate, AfS171, was chosen for further analyses.

*Chromatin immunoprecipitation (ChIP) protocol for identification of MetR targets.*

Overnight cultures of strains ATCC 46645 (control) and AfS171 [*metR::GA5::gfp2-5*] (tester) in *Aspergillus* minimal medium (AMM) containing methionine as S-source were shifted to AMM lacking any source of sulphur and incubated further at 37°C for 2 hours. Crosslinking was executed by addition of formaldehyde to a final concentration of 1% followed by incubation at 37°C for additional 20 minutes. Excess formaldehyde was quenched for five minutes by the addition of glycine to a final concentration of 125 mM. Mycelia were filtered and washed extensively with ice-cold phosphate-buffered saline (PBS), squeeze-dried, and snap-frozen in liquid nitrogen.

Frozen material was ground in liquid nitrogen and extracted with buffer S (50mM HEPES/KOH pH 7.5, 140mM NaCl, 1mM EDTA pH 7.5, 0,1% Triton X-100, 0.1% sodium deoxycholate) containing the cOmplete Ultra protease inhibitor cocktail (Roche). The resulting suspensions were sonicated to shear the chromatin to fragments with a medium lengths of 500 bp, as verified after agarose gel electrophoresis, followed by removal of the cell debris by centrifugation. The collected supernatants served as input for immunoprecipitation with the GFP-Trap technology (ChromoTek). In detail, aliquots of pre-equilibrated agarose beads coupled to the GFP-binding protein together with the sheared chromatin solutions were incubated at 4°C overnight under constant agitation. The supernatant was removed and beads were washed two times with Wash buffer (10 mM Tris/HCl pH 7.5, 150 mM NaCl, 0.5 mM EDTA), before precipitated fragments were eluted in 200 mM glycine (pH 2.5). After neutralization with 1 M Tris base (pH 10.4) and addition of NaCl to 1 M, proteins were digested by proteinase K at 42°C for 45 min and crosslinks were removed by overnight incubation at 65°C. DNA fragments were precipitated after phenol extraction and resuspended in 5 mM Tris/HCl (pH 8.5). Semi-quantitative PCRs to query enrichment for specific candidate promoter fragments were carried out with pairs of oligonucleotides spanning regions of ca. 200 bp and using equal amounts (20 ng) of template and retrieving samples after 25, 30, 35, and 40 cycles of amplification. ChIP samples were analyzed from two biological replicates to validate reproducible results.
